# A *Becn1* mutation mediates hyperactive autophagic sequestration of amyloid oligomers and improved cognition in Alzheimer's disease

**DOI:** 10.1371/journal.pgen.1006962

**Published:** 2017-08-14

**Authors:** Altea Rocchi, Soh Yamamoto, Tabitha Ting, Yuying Fan, Katherine Sadleir, Yigang Wang, Weiran Zhang, Sui Huang, Beth Levine, Robert Vassar, Congcong He

**Affiliations:** 1 Department of Cell and Molecular Biology, Feinberg School of Medicine, Northwestern University, Chicago, IL, United States of America; 2 Department of Microbiology, Sapporo Medical University School of Medicine, Sapporo, Japan; 3 Center for Autophagy Research, Department of Internal Medicine, Howard Hughes Medical Institute, University of Texas Southwestern Medical Center, Dallas, TX, United States of America; 4 Key Laboratory on Chemistry and Biology of Changbai Mountain Natural Drugs, School of Life Sciences, Northeast Normal University, Changchun, Jilin, China; 5 School of Life Sciences, Zhejiang Sci-Tech University, Hangzhou, Zhejiang, China; UCSD, UNITED STATES

## Abstract

Impairment of the autophagy pathway has been observed during the pathogenesis of Alzheimer’s disease (AD), a neurodegenerative disorder characterized by abnormal deposition of extracellular and intracellular amyloid β (Aβ) peptides. Yet the role of autophagy in Aβ production and AD progression is complex. To study whether increased basal autophagy plays a beneficial role in Aβ clearance and cognitive improvement, we developed a novel genetic model to hyperactivate autophagy in vivo. We found that knock-in of a point mutation F121A in the essential autophagy gene Beclin 1/*Becn1* in mice significantly reduces the interaction of BECN1 with its inhibitor BCL2, and thus leads to constitutively active autophagy even under non-autophagy-inducing conditions in multiple tissues, including brain. *Becn1*^F121A^-mediated autophagy hyperactivation significantly decreases amyloid accumulation, prevents cognitive decline, and restores survival in AD mouse models. Using an immunoisolation method, we found biochemically that Aβ oligomers are autophagic substrates and sequestered inside autophagosomes in the brain of autophagy-hyperactive AD mice. In addition to genetic activation of autophagy by *Becn1* gain-of-function, we also found that ML246, a small-molecule autophagy inducer, as well as voluntary exercise, a physiological autophagy inducer, exert similar *Becn1*-dependent protective effects on Aβ removal and memory in AD mice. Taken together, these results demonstrate that genetically disrupting BECN1-BCL2 binding hyperactivates autophagy in vivo, which sequestrates amyloid oligomers and prevents AD progression. The study establishes new approaches to activate autophagy in the brain, and reveals the important function of *Becn1*-mediated autophagy hyperactivation in the prevention of AD.

## Author summary

Accumulation of amyloid β peptides (Aβ) is a major cause of Alzheimer’s disease (AD). Although many efforts have been made, no effective therapies are available to cure AD. Autophagy is a stress-induced pathway nerve cells use to dispose damaged structures, and may be a strategy to eliminate Aβ aggregation. However, direct evidence of autophagic disposal of Aβ is lacking. Here we described a new AD mouse model that shows high autophagy activity even under non-autophagy-inducing conditions. In these mice, we engineered a single mutation into a key autophagy gene *Becn1*, which disrupts an inhibitory binding and leads to constitutively active autophagy in brain. Compared to regular AD mice, the autophagy-hyperactive AD mice are protected from amyloid accumulation, memory deficits and high mortality. We also isolated autophagic vesicle from these mice by an antibody, and found that Aβ oligomers are incorporated inside the vesicles. Thus, we obtained direct evidence for the first time that oligomerized amyloids are substrates of autophagy. These findings are important, as we demonstrated the reversal of AD progression by modulating the activity of a single autophagy gene. Our findings also revealed the therapeutic potential of brain-permeable autophagy-inducing chemicals in the prevention of AD by reducing intracellular amyloids.

## Introduction

Alzheimer’s disease (AD) is a neurodegenerative disorder characterized by protein aggregation and deposition, leading to progressive neuronal loss and cognitive decline among elderly populations [1]. Amyloid plaques and neurofibrillary tangles are the two primary hallmarks of AD pathology, and aging is a major known risk factor of the disease [2, 3]. Amyloid plaques are formed by amyloid-β (Aβ) peptides, generated by sequential enzymatic cleavages of amyloid precursor protein (APP) at the plasma membrane [4, 5]. Besides the well-recognized extracellular deposition of Aβ, recent studies also revealed the accumulation of intracellular pools of Aβ in AD brain. Intracellular Aβ can be generated at the trans-Golgi network and endoplasmic reticulum as part of the secretory pathway, or be reuptaken by neurons and glial cells from the secreted extracellular pools [6, 7]. Although many therapeutic efforts have been made to eliminate Aβ aggregation and deposition at either the synthesis or the degradation stage, no effective therapies are available so far to cure AD, and the mechanism driving the neurodegenerative progression remains unclear [8].

Autophagy is an evolutionarily conserved lysosomal catabolic pathway regulated by autophagy-related (ATG) proteins [9, 10]. Autophagy is induced by stress conditions such as hypoxia, starvation or oxidative stress [11, 12]; upon autophagy induction, autophagosomes sequester cytoplasmic components and fuse with lysosomes to generate autolysosomes, in which degradation of the autophagic cargos occurs [13, 14]. Although many studies have reported the roles of autophagy in the elimination of wasteful components, including protein aggregates, the relationship between autophagy and AD progression is complex. Several lines of evidence suggest an impairment of the autophagy pathway in the pathogenesis of AD. Brain from AD patients shows an abnormal accumulation of autophagic vacuoles and a reduction in the level of Beclin 1/BECN1, an essential autophagy protein and ortholog of ATG6 [15, 16]. However, direct evidence of autophagosome-mediated degradation of Aβ or APP in brain is lacking. Paradoxically, autophagy has been reported to promote, rather than reduce, the production of Aβ. Knockout (KO) of an essential autophagy gene *Atg7* specifically in forebrain excitatory neurons of AD mice decreases extracellular amyloid plaque formation, which is due to reduced processing and secretion of Aβ; however, these *Atg7* KO mice have exacerbated memory deficits [17], suggesting that the intracellular level of amyloids, which may be regulated by autophagy, may play a key role in cognitive impairment in AD. It is also under debate whether the level of the precursor protein APP is directly regulated by autophagy in either rodent brain or primary neurons [16–19]. On the other hand, enhancing lysosomal degradation capacity by genetic deletion of Cystatin B, a suppressor of lysosomal cysteine proteases, or use of autophagy-inducing chemicals such as a phytochemical Rg2 or the mTOR inhibitor rapamycin, reduces amyloid burden and memory deficit in mouse models of AD [20, 21, 22]. However, the mechanism of these compounds remains enigmatic. In addition, although knockout of autophagy genes leads to neurodegeneration [15, 23, 24], it is unknown whether physiologically increased basal autophagy prevents neurotoxicity of Aβ and has beneficial effects in protecting against Alzheimer’s-like diseases.

Thus, to directly assess the function of physiological enhancement of autophagy in vivo, we generated and characterized a unique mouse model of constitutively active autophagy caused by a single knockin mutation (F121A) in *Becn1*. We crossed these autophagy-hyperactive mice with the 5XFAD transgenic AD mice, which overexpress a combination of 5 familial Alzheimer’s disease (FAD) mutations in human APP and human PS1 (presenilin 1) proteins and show early amyloid deposition beginning at 2 months of age and cognitive decline at 6 months of age [25]. We demonstrated that elevated basal autophagy targets Aβ oligomers, and significantly reduces the accumulation of Aβ, but not APP. Genetic hyperactivation of autophagy also ameliorates neuronal dysfunction and enhances survival in AD mice. In addition to genetic activation of autophagy, we also found that autophagy hyperactivation either pharmacologically by a novel compound ML246 or physiologically by voluntary exercise protects AD mice from amyloid deposition and memory loss. Overall, this study provides the first evidence that hyperactive autophagy caused by a single mutation in *Becn1* sequesters amyloids and restores memory in AD, and also establishes the first genetic model of constitutively active autophagy as a useful in vivo tool to study autophagy in different diseases.

## Results

### A knockin point mutation F121A in *Becn1* leads to constitutively high autophagy in vivo

**Fig 1:**
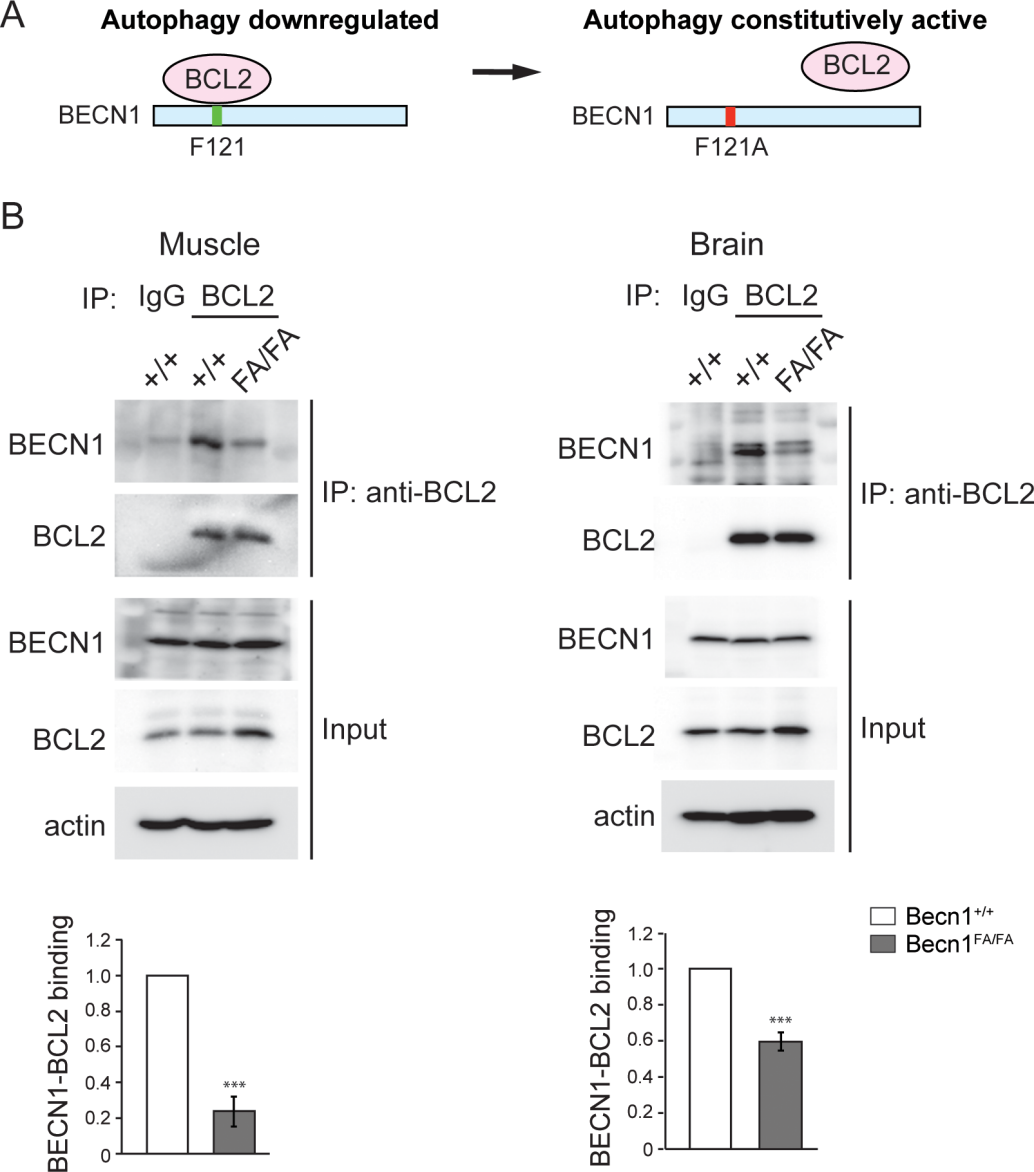
The *Becn1* F121A mutation inhibits the BECN1-BCL2 interaction in vivo. **(A)** Schematic representation of the strategy for hyperactive autophagy via the *Becn1*^F121A^ knockin allele. F121A blocks binding of BECN1 with its inhibitor BCL2, which leads to upregulated BECN1 function and constitutively high autophagy. **(B)** Co-immunoprecipitation of BECN1 by BCL2 in skeletal muscle and brain tissues from wild-type (WT) and *Becn1*^F121A^ mice. Less BECN1^F121A^ is immunoprecipitated by BCL2 than WT BECN1, quantified by the BECN1/BCL2 ratio in the IP samples from 3 independent experiments. FA/FA, *Becn1*^F121A^ homozygous knock-in mice. ***, P<0.001, t test.

To study how autophagy physiologically regulates the progression of Alzheimer’s disease (AD), we generated a new knock-in mouse model with hyperactive autophagy, by genetically disrupting the nutrient-regulated interaction between BECN1 and its inhibitor BCL2 (Fig 1A). Reversible BECN1-BCL2 binding is an important regulatory mechanism of autophagy induction [26]. When nutrients are abundant, BECN1 is bound and inhibited by BCL2, an anti-apoptotic and anti-autophagy protein. In response to stress such as starvation, BECN1 is released from the inhibitory binding of BCL2 for autophagy function [27, 28]. The BCL2 binding site in human BECN1 is reported as F123 [27]. We found that F121 in the BH3 domain of mouse BECN1 is the corresponding conserved residue of human F123. Thus, we proposed that mutating the residue F121 (TTT) to an alanine (A, GCT) disrupts BECN1-BCL2 binding and leads to constitutive activation of BECN1 and autophagy in mice (Fig 1A). We then generated a global knock-in mouse line (*Becn1*^FA/FA^) (S1A–S1C Fig), and found that the homozygous *Becn1*^FA/FA^ mice are viable, fertile, of normal size and weight, and display normal histology in major organs under normal housing conditions. The BECN1 protein expression level in *Becn1*^FA/FA^ mice is also comparable to that in WT mice in multiple major organs, including brain, heart, skeletal muscle, liver and pancreas (S2 Fig). Co-immunoprecipitation analysis showed that in *Becn1*^FA/FA^ mice, there is much less interaction between BECN1 and BCL2 in both skeletal muscle and brain than in WT mice (Fig 1B), suggesting that the F121A mutation significantly weakens BCL2 binding to BECN1 in vivo.

**Fig 2:**
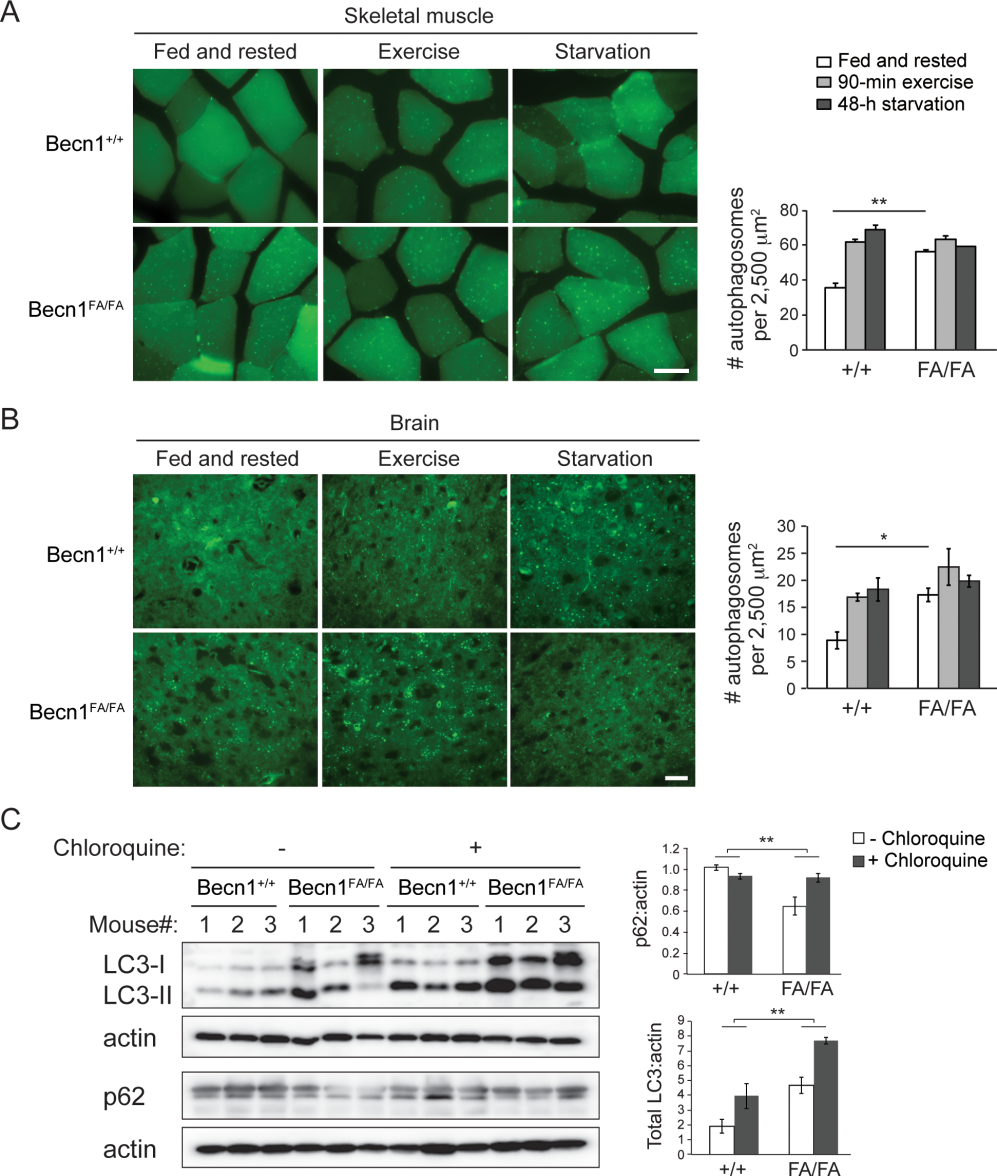
*Becn1*^F121A^ leads to autophagy hyperactivation in vivo. **(A, B)** Representative images (left panel) and quantification (right panel) of GFP-LC3 puncta (autophagosomes) in skeletal muscle **(A)** and brain **(B)** of GFP-LC3 *Becn1*^+/+^ and GFP-LC3 *Becn1*^FA/FA^ mice at non-autophagy-inducing conditions (fed and rested), after 90-min exercise, or after 48 hours of starvation. Scale bar: 25 μm. Results represent mean ± s.e.m. N = 5. *, P<0.05, **, P<0.01, t test. **(C)** Western blot analysis (left panel) and quantification (right panel) of LC3 and p62 levels in skeletal muscle from *Becn1*^+/+^ and *Becn1*^FA/FA^ mice injected with one dose of PBS or 50 mg/kg lysosomal inhibitor chloroquine. The autophagy flux is measured by the difference in the p62 and LC3 levels between mice injected with PBS and with chloroquine. Results represent mean ± s.e.m. N = 3. **, P<0.01, two-way ANOVA for comparison of magnitude of changes between different groups in mice of different genotypes.

To determine whether these mice have hyperactive autophagy, we crossed them with the GFP-LC3 autophagy reporter mice [29]. Upon autophagy induction, diffusely distributed autophagosome marker protein LC3 (LC3-I) is conjugated to phosphatidylethanolamine to form lipidated LC3 (LC3-II), which specifically associates with autophagosomal membranes and can be resolved by western blot or visualized as fluorescent puncta. We found that under non-autophagy-inducing conditions (fed and resting), *Becn1*^FA/FA^ knock-in mice exhibit a higher number of GFP-LC3 puncta (autophagosomes) in both skeletal muscle (Fig 2A) and brain (Fig 2B) than wild-type (WT) mice, which reaches similar levels under autophagy-inducing conditions (90-min treadmill exercise or 48-h starvation). These data suggest that *Becn1*^FA/FA^ mice show high basal autophagy. To determine that the increment of autophagosomes in *Becn1*^FA/FA^ mice is due to elevated autophagic flux, rather than a block in autophagosome degradation, we analyzed the autophagy flux by inhibiting lysosomal degradation using the lysosomal inhibitor chloroquine. In skeletal muscle of *Becn1*^FA/FA^ mice, chloroquine injection led to more accumulation of LC3 and GFP-LC3 puncta compared to WT mice, measured by western blot analyses and microscopy, respectively (Fig 2C and S3 Fig). In addition, compared to WT mice, *Becn1*^FA/FA^ mice showed a lower level of p62, an autophagy cargo protein, in skeletal muscle, which was rescued by chloroquine treatment (Fig 2C). These data suggest that the *Becn1*^F121A^ mutation in mice leads to higher autophagic degradation of LC3 and p62. Altogether, we conclude that mutating F121 to A disrupts BECN1-BCL2 binding and constitutively activates autophagy in mice, thus providing a novel mouse model with hyperactive autophagy as a useful tool to analyze the physiological effects of autophagy upregulation in vivo.

### *Becn1* F121A decreases amyloid accumulation and improves cognitive function in the 5XFAD Alzheimer’s mouse model

**Fig 3:**
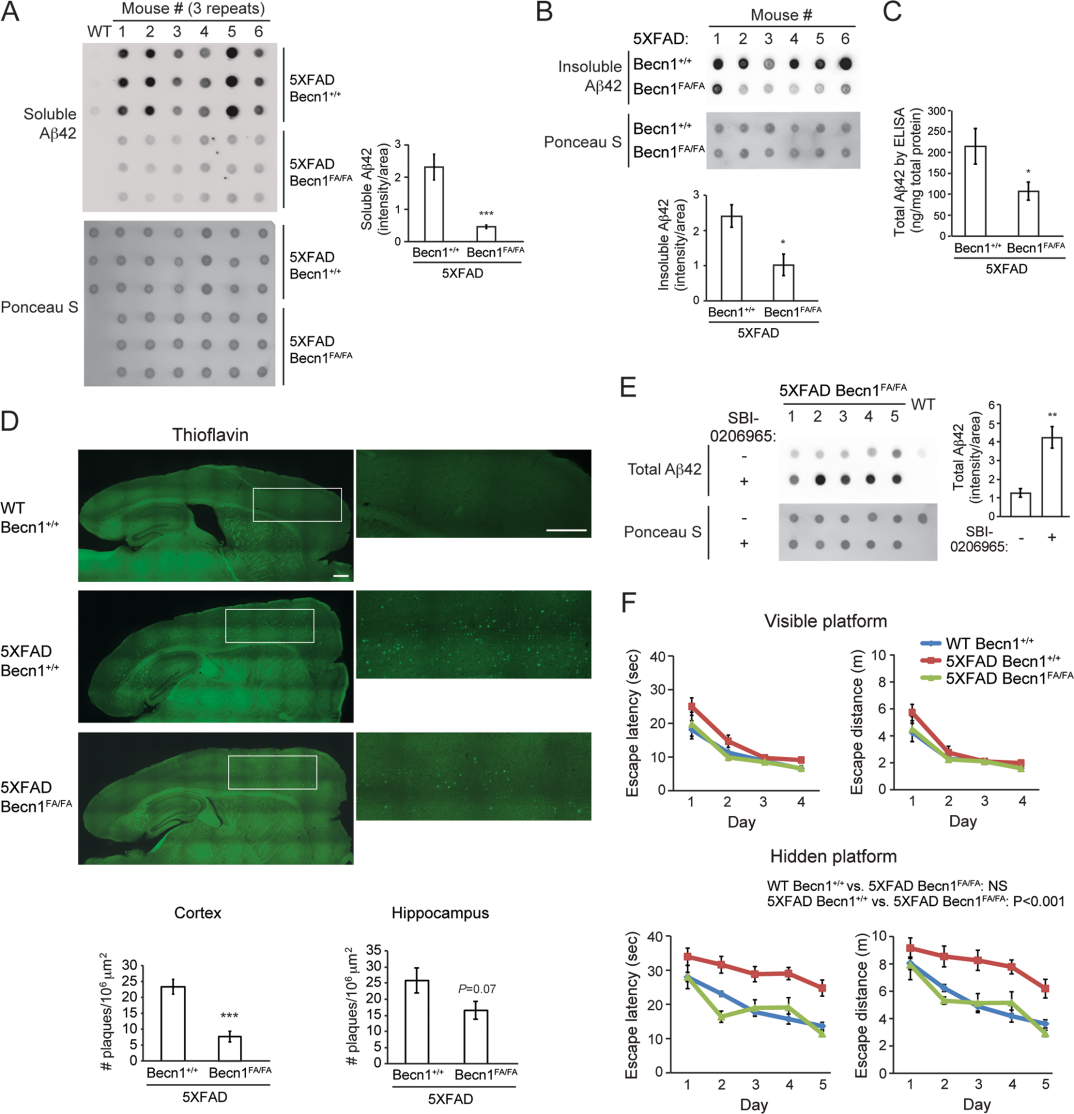
*Becn1*^FA/FA^ mutation ameliorates cerebral Aβ accumulation, memory deficits and mortality of Alzheimer’s mouse models. **(A-B)** Dot-blot assays and quantification of soluble **(A)** and insoluble **(B)** Aβ42 levels in homogenated brain samples of 6-month old 5XFAD *Becn1*^+/+^ and 5XFAD *Becn1*^FA/FA^ mice, immunostained with anti-Aβ42 antibody. Total protein loading was labeled by Ponceau S. Triplicate experiments from 6 mice in each group were shown. ***, P<0.001, t test. **(C)** ELISA analyses of total (soluble and insoluble) Aβ42 levels in the cortex of 6-month old 5XFAD *Becn1*^+/+^ and 5XFAD *Becn1*^FA/FA^ mice. N = 7. **(D)** Representative images (upper) and quantification (lower) of amyloid plaques stained by Thioflavin S in brain (cortex and hippocampus) of 5XFAD *Becn1*^+/+^ and 5XFAD *Becn1*^FA/FA^ mice. Magnification of the enclosed regions is shown on the right. N = 6–8. Scale bar: 500 μm. ***, P<0.001, t test. **(E)** Dot-blot assays and quantification of total Aβ42 levels in homogenated brain samples of 6-month old 5XFAD *Becn1*^FA/FA^ mice treated with the autophagy inhibitor SBI-0206965 or vehicle once per day for 7 days. Total protein loading was labeled by Ponceau S. N = 5. **, P<0.01, t test. **(F)** Morris water maze test of 6-month old WT, 5XFAD *Becn1*^+/+^ and 5XFAD *Becn1*^FA/FA^ mice. Escape latency and total distance traveled in visible platform test and hidden platform test are shown. N = 11–20. Results represent mean ± s.e.m. Two-way repeated measures ANOVA.

To determine the effects of autophagy activation on AD, we crossed the *Becn1*^F121A^ mice with the 5XFAD mice, an amyloid mouse model used in AD research. 5XFAD mice demonstrate early and aggressive phenotypes of intraneuronal Aβ42 aggregates, β-amyloid plaques and neurodegeneration, and represent a good model for our study. We analyzed the amyloid burden in the resulting 5XFAD *Becn1*^FA/FA^ mice by dot blot assays, ELISA and microscopy at the age of 6 months (Fig 3A–3D). The dot blot assay has been previously validated to measure levels of Aβ42 in APP transgenic mouse [30]. We found that 5XFAD *Becn1*^FA/FA^ mice show lower levels of both soluble and insoluble Aβ42 in the brain than the 5XFAD mice by dot blot assays (Fig 3A and 3B), whereas expression of the precursor APP remained unaffected (S4A Fig), suggesting that *Becn1*^FA/FA^-mediated hyperactive autophagy downregulates the levels of Aβ42, but not of APP. This is also confirmed by ELISA analyses on the level of total brain Aβ42 (Fig 3C). Furthermore, staining of amyloid plaques by Thioflavin S (Fig 3D) or Aβ42 antibody (S7A Fig) showed that there is a significant reduction of amyloid plaques in the cortex and a trend of reduction in the hippocampus of 5XFAD *Becn1*^FA/FA^ mice.

Importantly, *Becn1*^F121A^-induced reduction in Aβ42 is dependent on autophagy but not other pathways that regulate amyloid transport. We found that short-term (7-day) treatment of 5XFAD *Becn1*^F121A^ mice with SBI-0206965, an autophagy inhibitor blocking the kinase activity of an essential upstream kinase ULK1 [31], abolished the reduction in brain Aβ42 levels by dot blot assays (Fig 3E). Similarly, in HEK293 cells stably expressing APP and *Becn1*^F121A^, siRNA knockdown of the essential autophagy gene *ATG7* significantly increased the level of intracellular Aβ42 by western blot analysis (S5A Fig). The reduced Aβ42 level is not due to alterations in APP trafficking in *Becn1*^F121A^ mice, as immunofluorescence microscopy showed no detectable difference in the amount of APP colocalized with Rab5+ early endosomes or Rab7+ late endosomes in primary cortical neurons isolated from PDAPP *Becn1*^+/+^ mice and PDAPP *Becn1*^FA/FA^ mice (PDAPP mice is another amyloid model as described below) (S5B Fig). Furthermore, we also biochemically analyzed APP internalization and trafficking, by biotin protection assays using HEK293 cells stably expressing APP and WT *Becn1* or *Becn1*^F121A^ (the endogenous *BECN1* was deleted by CRISPR/Cas9) (S5C Fig). After inducing endocytic trafficking of cell surface APP by incubating the cells at 37°C for 5 min or 15 min, we observed a similar level of APP endocytosis (represented by biotinylated APP that is protected from glutathione stripping) in cells expressing WT *Becn1* and expressing *Becn1*^F121A^. Thus, altogether, these results demonstrated that *Becn1*^F121A^ does not affect APP trafficking. In addition, 5XFAD *Becn1*^F121A^ mice expressed a similar level of amyloid receptors in the brain that contribute to the clearance of Aβ, such as LDLR (Low-density lipoprotein receptor) and LRP1 (LDLR-related protein 1), compared to 5XFAD mice expressing WT *Becn1* (S5D Fig). Thus, altogether, we conclude that the *Becn1*^F121A^ knockin mutation reduces amyloid accumulation, and the effect of *Becn1*^F121A^ on Aβ metabolism is mediated by the hyperactive autophagy activity.

Next, to analyze memory function, we performed Morris water maze tests on WT mice and AD mice with normal or high autophagy. During the visible platform training, all 3 groups of mice showed no significant difference in either escape latency or distance (Fig 3F), suggesting that there was no visual or swimming abnormality among all groups. In contrast, during the hidden platform trials, 5XFAD mice expressing WT *Becn1* showed apparent deficiency in memorizing the platform location, while 5XFAD *Becn1*^FA/FA^ mice had significantly improved performance day by day in both escape latency and distance, similar to WT mice (Fig 3F). These data suggest that the memory impairment caused by Aβ accumulation is ameliorated by the *Becn1* F121A mutation. Overall, we conclude that genetic stimulation of basal autophagy mediated by *Becn1*^F121A^ reduces Aβ42 levels and plaque formation in mouse brain, and improves memory capacity that is impaired by amyloid aggregation in AD.

### *Becn1*^F121A^ increases survival of PDAPP AD mice

**Fig 4:**
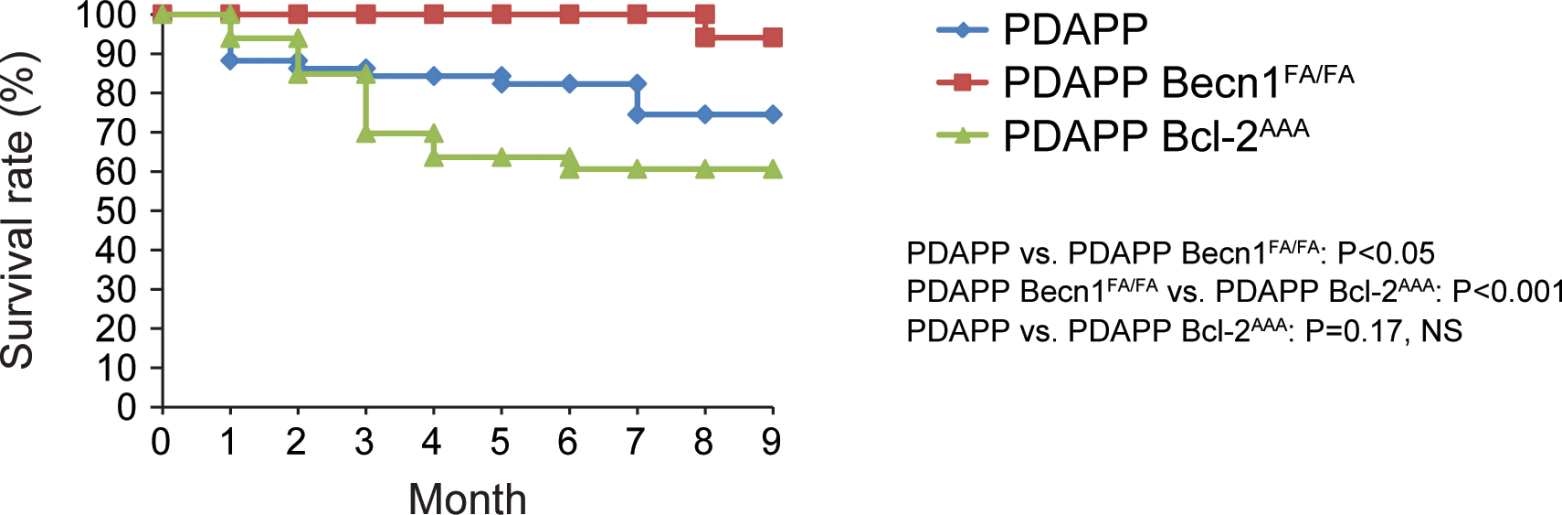
*Becn1*^F121A^ improves the survival rate of PDAPP AD mice. Kaplan-Meier survival curve of PDAPP mice with normal (PDAPP, N = 51), hyperactive (PDAPP *Becn1*^FA/FA^, N = 34), or deficient (PDAPP *Bcl2*^AAA^, N = 33) autophagy monitored over time for 9 months. Statistical significance was analyzed by the log-rank test.

To fully analyze the function of the *Becn1*^F121A^ allele in AD, we used another amyloid mouse model, known as PDAPP mice. These mice carry a V717F (Indiana) mutation in APP [32], and exhibit extracellular amyloid deposition starting at 6–9 months of age. The PDAPP mice have been shown to display an increased mortality rate compared to other AD lines [33, 34]. Similar to previous reports, we found that PDAPP mice have higher early mortality than WT mice starting at 2 months of age (Fig 4). We crossed PDAPP mice with either the autophagy-hyperactive *Becn1*^FA/FA^ mice, or autophagy-deficient *Bcl2*^AAA^ mice. *Bcl2*^AAA^ mice contain 3 knock-in alanine mutations (T69A, S70A and S84A) at the phosphorylation residues of BCL2, which block BCL2 phosphorylation and BECN1 release from BCL2 binding; thus, they are opposite to the *Becn1*^F121A^ mice and show defective autophagy [35]. Notably, homozygous expression of the *Becn1*^F121A^ mutation decreased mortality in PDAPP mice, while the autophagy-deficient PDAPP *Bcl2*^AAA^ mice showed a trend of exacerbated mortality compared to the PDAPP mice with normal autophagy (Fig 4). These data suggest a positive impact of hyperactive autophagy mediated by *Becn1*^F121A^ on the survival of PDAPP Alzheimer’s mice.

### Aβ oligomers are sequestered inside autophagosomes

**Fig 5:**
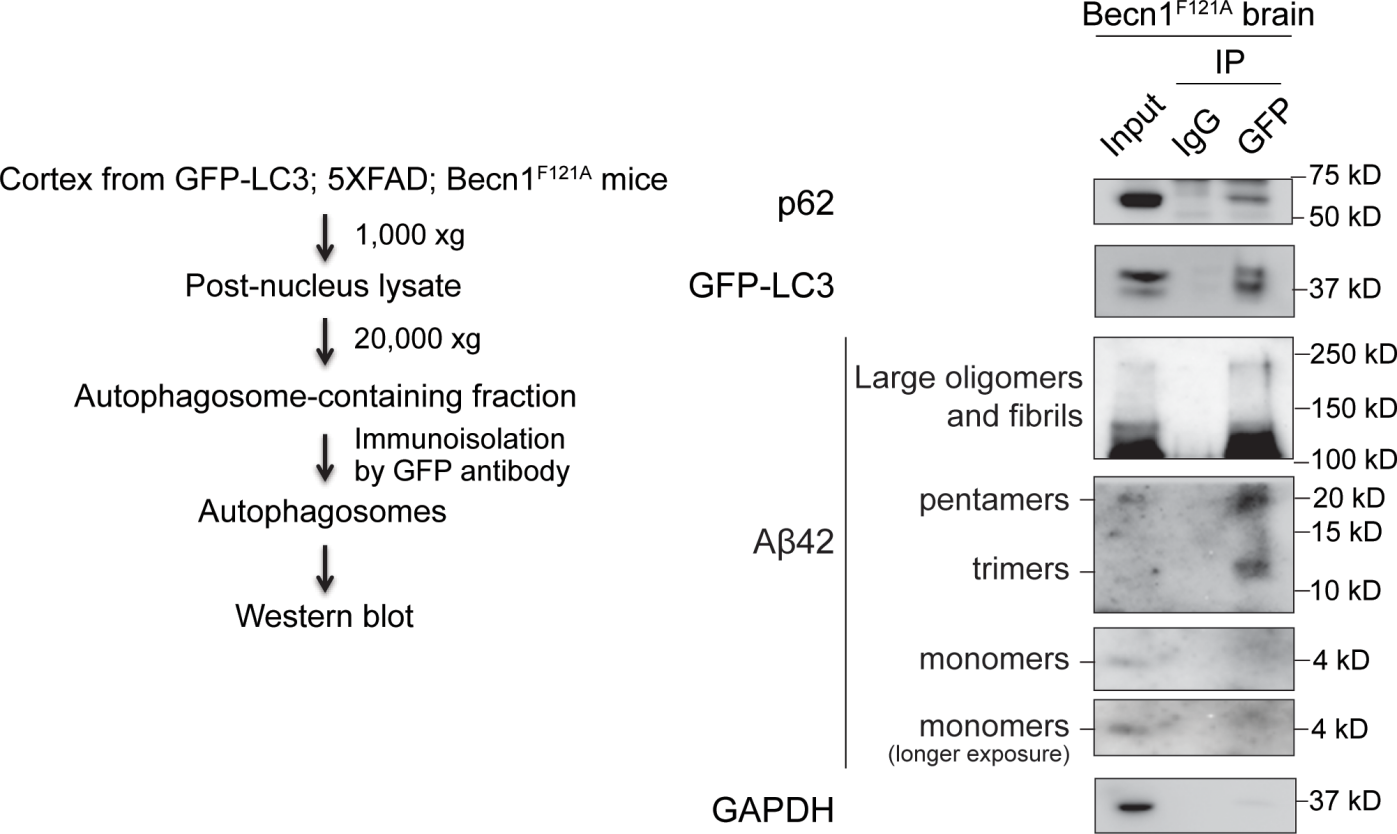
Autophagosomal sequestration of Aβ42 in brain of autophagy-hyperactive mice. (Left) Scheme of immunoisolation of autophagosomes from brain of 12-week old 5XFAD *Becn1*^FA/FA^ mice expressing GFP-LC3. Briefly, post-nucleus extracts of the brain lysates was obtained by centrifugation at a low speed of 1,000 xg. Autophagosomes were enriched by centrifugation at a high speed of 20,000 xg, and pulled down by an anti-GFP antibody using magnetic beads. (Right) Western blot detection of Aβ42 fibrillar and oligomeric species inside autophagosomes immunoprecipitated by GFP antibody as in the scheme. A known autophagy cargo p62 serves as a positive control, and a cytosolic enzyme GAPDH is a negative control.

To directly address whether intracellular amyloids are efficient autophagic cargos, and degraded by the autophagy machinery upon autophagy hyperactivation, we developed a method to immunoisolate intact autophagosomes from the cortex of 5XFAD *Becn1*^FA/FA^ mice expressing the autophagosome marker GFP-LC3. After sequential centrifugation and immunoprecipitation by anti-GFP antibody and magnetic beads, the purity of autophagosomes was validated by co-isolation of a known autophagy cargo p62 but not a cytosolic enzyme GAPDH (Fig 5). We found that Aβ42 oligomers, including trimers, pentamers and higher-molecular weight fibrils or fibril intermediates (of size between 100 kD and 250 kD), but not monomers, are co-immunoprecipitated and concentrated with autophagosomes from autophagy-hyperactive mice (Fig 5). Thus, these data suggest biochemically that intracellular Aβ oligomers are cargos of autophagy, and are sequestered and cleared by hyperactive autophagy in brain.

### Autophagy stimulation by ML246 and voluntary exercise reduces Aβ accumulation and improves cognitive function in 5XFAD mice

**Fig 6:**
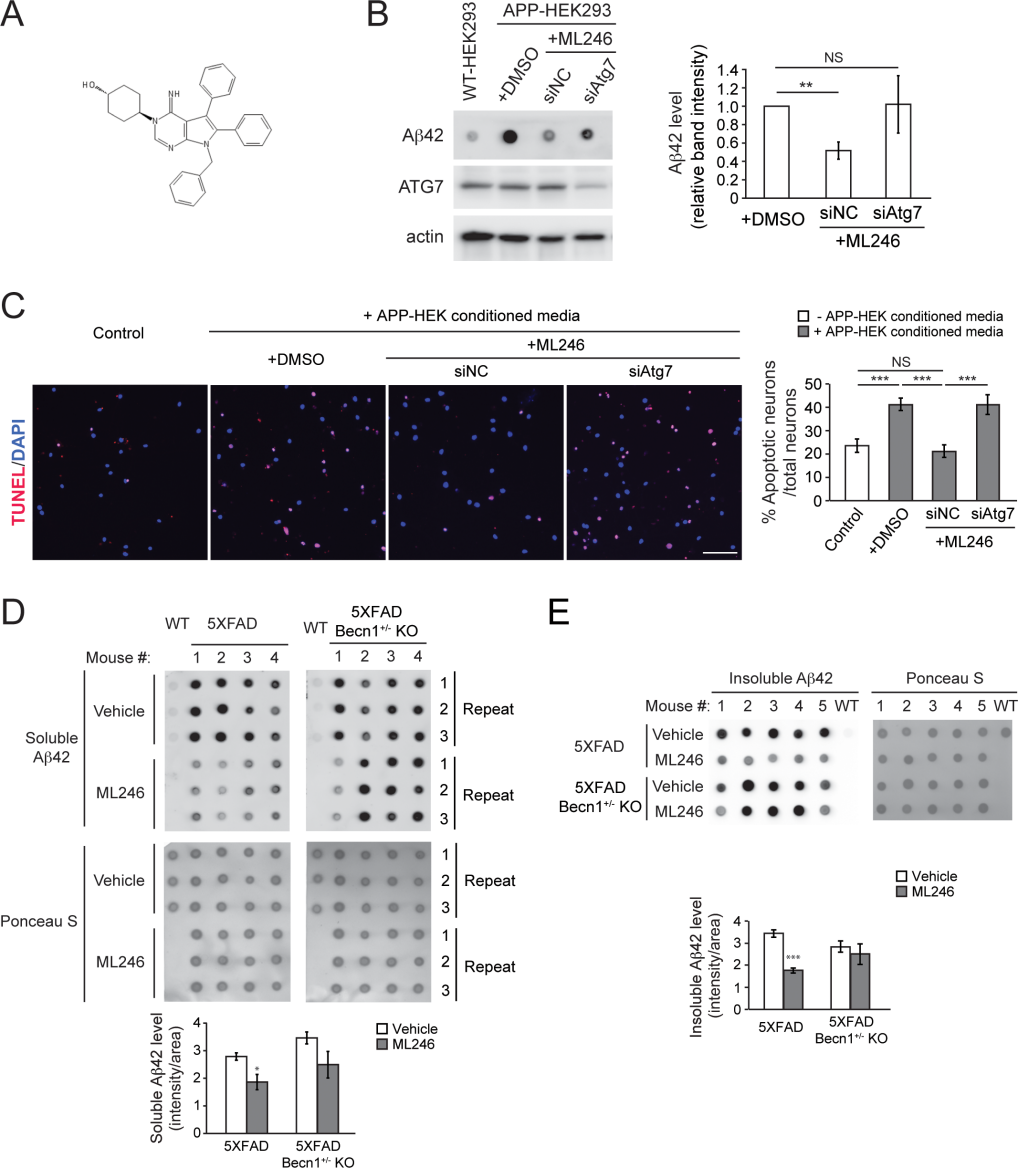
An autophagy-inducing compound ML246 reduces amyloid load in an autophagy-dependent manner in vitro and in vivo. **(A)** Chemical structure of ML246. **(B)** Dot-blot assays (left) and quantification (right) of secreted Aβ42 levels in conditioned media of HEK293 cells stably expressing APP treated with vehicle (DMSO) or ML246 for 24 h, immunostained with anti-Aβ42 antibody. Cells were transfected with non-targeting control (NC) or *ATG7* siRNA 24 h prior to ML246 treatment. Results are quantified from 4 independent experiments. **(C)** Representative images (left) and quantification (right) of TUNEL signals (red) in WT primary cortical neurons treated with conditioned media from (**B**) for 24 h. Nuclei were stained with DAPI. Scale bar, 100 μm. N = 10 fields (each field containing 20–30 neurons). **(D, E)** Representative images (upper) and quantification (lower) of dot-blot assays on soluble **(D)** and insoluble **(E)** Aβ42 levels in brain samples of 6-month old 5XFAD and 5XFAD *Becn1*^+/-^ KO mice after 5 weeks of ML246 treatment, immunostained with anti-Aβ42 antibody. Total protein loading was labeled by Ponceau S. Triplicate experiments from 4–5 mice in each group were shown. Results represent mean ± s.e.m. NS, not significant; *, P<0.05; **, P<0.01; ***, P<0.001, t test.

In addition to *Becn1*^F121A^-mediated genetic activation of autophagy, we decided to further study whether stimulating autophagy pharmacologically is also protective against neurodegenerative progression. We recently identified a brain-penetrable autophagy-inducing small molecule ML246 (metarrestin)[36] (Fig 6A), and analyzed its effects on the clearance of aggregate-prone proteins in vitro and in vivo. For in vitro analyses, we utilized the HEK293 cell line stably expressing APP (APP-HEK293), in which the produced Aβ molecules are efficiently secreted, to study the effect of ML246 on amyloid metabolism. Via dot blot assays, we found that ML246 treatment for 24 h significantly reduced the level of secreted Aβ in the conditioned media (Fig 6B). In addition, cultured WT primary cortical neurons treated with the conditioned media from ML246-treated APP-HEK293 cells underwent a lower level of apoptotic cell death than those treated with media from vehicle-treated APP-HEK293 cells (Fig 6C). These results demonstrate that ML246 reduces amyloid production and secretion in vitro. Importantly, siRNA knockdown of the essential autophagy gene *ATG7* in APP-HEK293 cells reversed the ML246-mediated reduction of both secreted Aβ42 (Fig 6B) and apoptotic neuronal death (Fig 6C), suggesting that the effect of ML246 in amyloid metabolism is autophagy-dependent.

In addition to the amyloid cell model, we also found that ML246 promotes the removal of intracellular aggregates formed by polyglutamine (polyQ)-expansion proteins. We used HeLa cell lines stably expressing tetracycline-repressible expanded polyQ-repeat protein HTT (huntingtin) as a model, HTT65Q and HTT103Q [37]. In contrast to the HTT protein with the normal number of glutamine repeats (HTT25Q), HTT65Q and HTT103Q formed insoluble polyQ aggregates larger than 0.2-μm in diameter, which can be detected by filter trap assay. We discovered that the accumulation of both HTT65Q and HTT103Q aggregates is decreased after ML246 treatment for 24 h, whereas knockdown of *ATG7* prevents this reduction (S6A Fig), suggesting that ML246 reduces intracellular protein aggregation, and this effect is dependent on the autophagy activity. Fluorescence imaging further confirmed that ML246 administration decreased the number of cells positive for HTT aggregates, which is also in an ATG7-dependent manner (S6B Fig). Thus, these data indicate that the autophagy pathway stimulated by ML246 promotes the clearance of aggregate-prone proteins (including both amyloid and polyQ expansion proteins) in vitro, and ML246 can be used as a candidate compound for in vivo analyses in AD mouse models.

Accordingly, we investigated the function of ML246-induced autophagy in amyloid accumulation and cognitive function in 5XFAD mice. Via dot blot assays, we found that compared to the ones treated with vehicle, 6-month old 5XFAD mice treated with ML246 for 5 weeks showed decreased levels of both soluble (Fig 6D) and insoluble Aβ42 in brain (Fig 6E). The expression of the precursor APP was not affected (S4B Fig), supporting the hypothesis that the level of Aβ42, but not APP, is regulated by autophagy. Notably, the effect of ML246 was abolished in the autophagy-deficient 5XFAD *Becn1*^+/-^ KO mice (Fig 6D and 6E), further supporting that ML246-induced reduction of Aβ42 in vivo is autophagy-dependent.

**Fig 7:**
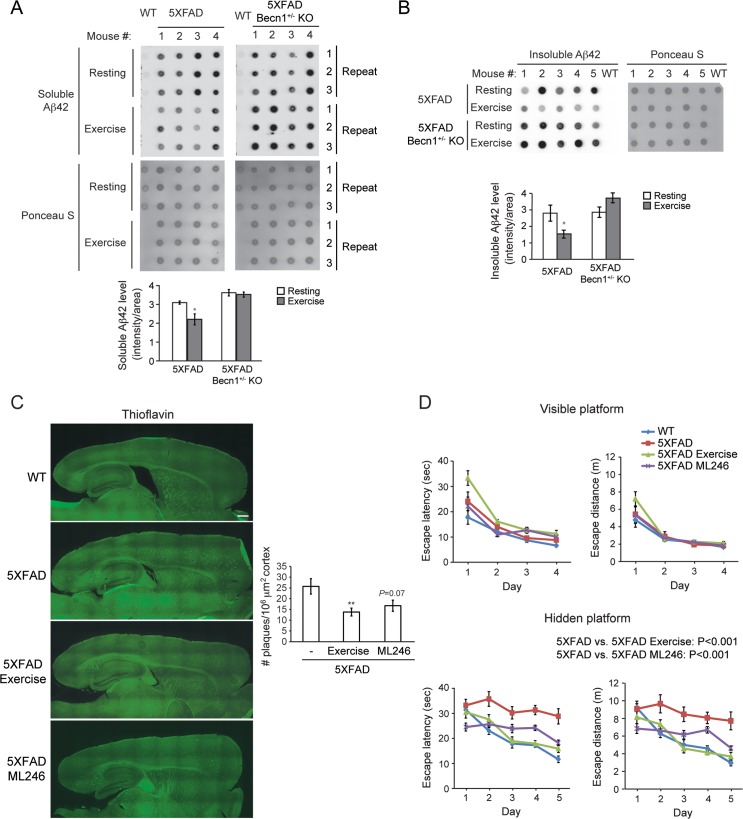
ML246 and voluntary exercise decrease cerebral amyloid plaques and ameliorate memory deficits in 5XFAD AD mice. **(A, B)** Representative images (upper) and quantification (lower) of dot-blot assays on soluble **(A)** and insoluble **(B)** Aβ42 levels in brain samples of 6-month old 5XFAD and 5XFAD *Becn1*^+/-^ KO mice after 4 months of voluntary running, immunostained with anti-Aβ42 antibody. Total protein loading was labeled by Ponceau S. Triplicate experiments from 4–5 mice in each group were shown. **(C)** Representative images (left) and quantification (right) of amyloid deposits stained by Thioflavin S in brain of 6-month old 5XFAD mice, and 5XFAD mice subject to 5 weeks of ML246 treatment or 4 months of voluntary exercise. Scale bar: 500 μm. Results represent mean ± s.e.m. N = 6–8. *, P<0.05; **, P<0.01, t test. **(D)** Morris water maze test of 6-month old WT, 5XFAD, and 5XFAD mice after 5 weeks of ML246 treatment or 4 months of voluntary running. Escape latency and total distance traveled in visible platform test and hidden platform test are shown. N = 10–11. Results represent mean ± s.e.m. Two-way repeated measures ANOVA.

Moreover, in addition to pharmacological approaches, physical exercise has recently been demonstrated as a fast and robust physiological method to induce autophagy in various tissues, including brain [38]. Intriguingly, previous studies indicated that aerobic exercise decreases amyloid load in AD mouse models [39–42], and is also associated with a lower risk of cognitive decline among elderly populations [43–46]. Thus, we hypothesized that exercise-induced autophagy may represent a cellular mechanism underlying the neuroprotective effects of exercise in AD brain. To test this hypothesis, we housed 2-month old 5XFAD mice individually with access to a running wheel for 16 weeks. Through dot blot assays on brain lysates, we found that 5XFAD mice subject to 16 weeks of voluntary running have significantly lower levels of both soluble (Fig 7A) and insoluble (Fig 7B) Aβ42 in brain than those housed under resting conditions (without running wheels), suggesting that physical exercise decreases the amyloid burden in AD mouse brain. Fluorescence microscopy also shows a significant reduction of amyloid plaques after exercise training, and a trend of plaque reduction after ML246 treatment, stained by thioflavin S or Aβ42 antibody in brain of 5XFAD mice, especially in the cerebral cortex (Fig 7C and S7A Fig). Similar to ML246 treatment, APP expression is not affected by exercise (S4C Fig). In comparison, exercise failed to reduce amyloid accumulation in the autophagy-deficient 5XFAD *Becn1*^+/-^ KO mice (Fig 7A and 7B), suggesting that the autophagy pathway is required for the effects of exercise on amyloid accumulation.

Finally, to analyze whether the autophagy-inducing compound ML246 has the potential to improve the cognitive function of Alzheimer’s mice, we performed Morris water maze tests on 6-month old 5XFAD mice injected with ML246 daily for 5 weeks. We found that ML246 treatment, as well as 16-week voluntary exercise, improved the performance of 5XFAD mice during the hidden platform trials, compared to the vehicle-treated resting mice at the same age (Fig 7D). These data suggest that similar to exercise, the autophagy inducer ML246 ameliorates memory impairment in AD mice.

## Discussion

The role of autophagy in amyloid production and clearance has been unclear. In this study, we generated a mouse model with hyperactive autophagy by knocking-in a point mutation F121A to Beclin 1/*Becn1*, and found that *Becn1*^F121A^-mediated autophagy hyperactivation reduces brain amyloid accumulation, ameliorates cognitive deficits, and improves survival rates in Alzheimer’s mouse models.

BECN1 is a core component of the type III phosphatidylinositol-3-kinase (PI3K) complex, and is key for the initiation of autophagosome biogenesis [26]. Lentiviral overexpression of *Becn1* has been shown to reduce APP levels in cultured CHO cells or decrease amyloid deposition in AD mouse brain [15, 16]. Yet it is unclear whether *Becn1* overexpression represents a physiological method for autophagy activation. Thus, we designed a strategy to constitutively activate autophagy in vivo by preventing BECN1 from binding with its inhibitor BCL2. Under nutrient rich conditions, BECN1 is bound and inhibited by BCL2; whereas in the presence of stress such as nutrient starvation and exercise, BCL2 is phosphorylated and released from BECN1, which activates autophagy [28] and represents a physiological regulatory mechanism of the function of *Becn1* in autophagy. In our new knock-in mouse model, the introduction of the F121A mutation in *Becn1* (F121A) disrupts the BCL2 binding site, resulting in the constitutive activation of BECN1 in autophagy that is no longer regulated by stress. In skeletal muscle and brain of the *Becn1*^F121A^ mice, the autophagy levels under basal conditions are as high as those obtained after physical exercise or starvation in WT mice. Thus, we consequently crossed these mice with amyloid mouse models, including 5XFAD and PDAPP mice, to study the function of *Becn1*-mediated autophagy in AD. Given the roles of autophagy in a broad spectrum of diseases, this new mouse model can be a useful genetic tool to study the physiological effects of autophagy hyperactivation in multiple diseases.

We found that *Becn1*^F121A^-mediated autophagy hyperactivation decreases Aβ levels and improves memory in 5XFAD mice. Yet how the autophagy pathway downregulates intracellular Aβ still remains mysterious. Besides the plasma membrane, APP also localizes to the secretory pathway (such as the trans-Golgi network and endoplasmic reticulum), endosomes, lysosomes and mitochondria. We do not know whether it is the intracellular Aβ, or the extracellular secreted pool taken back up by cells, that is regulated by autophagy in *Becn1*^FA/FA^ mice. Several studies also suggest that BECN1 promotes internalization and lysosomal trafficking of the precursor protein APP. In cultured neuronal and HEK293 cell lines, BECN1 has been reported to promote endocytosis and endolysosomal and autolysosomal proteolysis of plasma membrane APP [47]. The adaptor protein AP2 seems to interact with LC3 to target APP to autophagosomes [19]. However, whether APP trafficking and degradation depends on other key components in the autophagy machinery is not known, and whether the process of autophagosome-mediated APP degradation occurs in AD mouse brain or neurons is still under debate [18]. Our data argue against a role of autophagy in regulating the levels of APP, since we found that *Becn1*^F121A^ does not alter the level, internalization, or trafficking of APP in mouse brain, primary cortical neurons, or cell lines (S4 and S5B and S5C Figs), suggesting that the effect of *Becn1*^F121A^ on amyloid metabolism is not through the regulation of APP.

The role of autophagy in the regulation of Aβ is more complex. On one hand, *Becn1* has been shown to be important for the phagocytosis and autophagic degradation of extracellular Aβ by cultured microglial cells [48, 49], and *Becn1*-deficient mice showed impaired Aβ clearance [49], which is consistent with our findings. On the other hand, autophagy is suggested to facilitate Aβ processing and secretion from neurons, using neuroglioma cell lines [50] and tissue-specific *Atg7* KO mice in excitatory forebrain neurons [17]. Thus, our autophagy-hyperactive AD mouse model is useful to assess the overall readout of autophagy activation on Aβ levels in vivo (Figs 3 and 4). Using this model system, we biochemically detected Aβ oligomers in purified intact autophagosomes (Fig 5), suggesting that autophagy plays a direct role in brain amyloid clearance. We propose a model in which autophagic degradation of Aβ occurs in both neurons and glial cells, where neuronal autophagy mainly degrades de-novo processed Aβ, whereas autophagy in glia removes Aβ reuptaken from the extracellular space (S7B Fig). As future directions, it will be interesting to determine whether the endocytic reuptake and trafficking machinery in neurons or glia is required for autophagic degradation of Aβ, and to discover what receptors are involved in the autophagosomal recognition of Aβ42-containing secretory or endocytic vesicles.

Furthermore, besides hyperactivating autophagy by genetic factors, we analyzed the effects of ML246, a novel autophagy-inducing compound that can pass the blood-brain barrier [36], on Aβ accumulation and cognition in AD mice. Pharmacological strategies to modulate autophagy have been recently proposed in the prevention of neurodegenerative diseases [51]. Most autophagy inducers that have been tested are based on inhibiting the autophagy suppressor mTOR, such as the well-known mTOR inhibitor rapamycin [21, 52, 53], which seems to be effective to decrease Aβ levels and prevent cognitive impairment in AD mice when used at early stages prior to the formation of extracellular plaques. Here we showed that ML246 is able to decrease protein aggregates in cultured cells, and reduce Aβ levels and ameliorate memory deficit in 5XFAD mice, and notably, we started compound treatment at the age of 4–5 months when amyloid deposition has already been documented in this AD mouse model [25]. Thus, ML246 is a new autophagy activator of neuroprotective function and potential use in AD treatment, although the signaling pathways and mechanisms by which ML246 induces autophagy are yet to be determined.

In addition to genetic and compound inducers of autophagy, we also studied whether activating autophagy by physiological methods prevents AD. Starvation and exercise are the best-known physiological inducers of autophagy in vivo [29, 35, 54]. Interestingly, although starvation induces detectable formation of autophagosomes in neurons of 3-month 5XFAD mice after 48 h [55, 56], it seems ineffective in removing intra-neuronal or extracellular Aβ [56], likely due to insufficient degradation of Aβ-containing autolysosomes after short-term starvation. In comparison, exercise, either forced exercise by treadmill or voluntary exercise by running wheel, has been recently shown to increase the autophagy flux in various tissues, including skeletal muscle and cerebral cortex in mice [35, 38, 54, 57]. Thus, we investigated the effects of physical exercise in AD, and demonstrated that 4 months of voluntary running exerted positive effects on animal behavior and amyloid pathology in brain of 5XFAD mice. It should be noted that we started voluntary exercise at the age of 2 months prior to any detectable cognitive impairment. Physical exercise has previously been suggested to play a beneficial role against cognitive decline in AD [39, 41, 42], but the molecular mechanism remains unknown. We showed for the first time that compared with AD mice with normal autophagy activity, exercise is not able to reduce amyloid deposition in brain of autophagy-deficient AD mice, suggesting that exercise-induced autophagy may be an important mechanism mediating some of the beneficial effects of exercise on AD [35, 54], although exercise may affect other pathways that also contribute to the exercise-mediated neuroprotective effects. Intriguingly, treadmill exercise does not seem to be as effective as voluntary wheel running to prevent neurodegeneration [40]. It is likely that the stress associated with forced running on the treadmill exerts detrimental effects on animal behavior and disease pathogenesis. Finally, considering that one key problem in AD is the late diagnosis of the disease that significantly reduces the effectiveness of subsequent treatments [58, 59], voluntary exercise should be considered as an important component in modern lifestyle to effectively induce autophagy and prevent cognitive decline as a non-pharmacological intervention.

In summary, in this study we developed 3 new strategies to potently activate autophagy in the brain, genetic (by the *Becn1*^F121A^ mutation), pharmacological (by ML246), and physiological (by voluntary exercise). Using the different approaches, we provided evidence that autophagy induction ameliorates amyloid pathology and reduces cognitive deficits in 5XFAD mice. Our data revealed the potential of autophagy stimulation in lowering toxic aggregate-prone proteins and improving neuronal functions for the treatment of AD.

## Methods

### Mice

All animal experiments have been approved by the Northwestern University Institutional Animal Care and Use Committee (IACUC) (Protocol number: IS00004749). All mice were housed on a 12-h light/dark cycle, and male mice were used for behavioral analyses. All mice were in the C57BL/6 background except PDAPP mice. The PDAPP mice were generated in a C57BL/6 and DBA2 mixed genetic background and have been backcrossed with C57BL/6 mice for 8 generations prior to analyses. GFP-LC3 transgenic, *Becn1*^+/-^ KO, *Bcl2*^AAA^, PDAPP and 5XFAD mice have been previously described [25, 29, 32, 35, 60]. For the construction of a mouse strain with the F121A knock-in allele in *Becn1*, BAC clones (Incyte) were screened for the presence of *Becn1*. The *Becn1* BAC clone was subcloned into the pVB vector and the F121 (TTT) in exon 6 of *Becn1* was replaced by A (GCT). A neomycin resistance marker flanked by LoxP sites was inserted between exons 7 and 8. The resulting targeting construct, pVBKI-*Becn1*, was linearized by I-CeuI digestion and electroporated into 129 Sv/J × C57BL/6J hybrid ES cells, and 36 h later, clones were selected with neomycin, and screened by Southern blot analysis with the probes indicated in S1 Fig. DNA was digested with HindIII, and electrophoretically separated on a 0.8% agarose gel. After transfer to a nylon membrane, the digested DNA was hybridized with a probe targeted against the 5’ region (Probe 1):

TCCAGTGATGATGGTGGTGCTGATAATAATAGGGATGTTTTCATTACCAAAGATAGATGTTGTAGCTTGATTTTCTTTTGTGGGGAGCAGGTCATTGTCAAGTAGAAGTTACTGACTTGGGAGAGGATCCCAAGGGACCCTAGTACAAAATAGAGAAAACGGATGGGTGGAAAGGGAAAGAAGCCTAGGAGGGAGACATGGTCACACACCAGTGGCACAGCATCCTGGGGAAAGCGCTGGCCTCATCCCTGAGATTTACCTTGCCTGAGCAATACGGGAGGATTTATCCGAGTGACTGCTGTCACTGGGAAAAGCGAACCTTAAGTGGGTTGGGGGCTGTTAATTCTAGCATGCAAGGCCAGAGAAAACCTGCAAAGAAGCAAAAGAGGCAGGCAGCTGAAGCCAGTGTGTTCAAAATGTTGAACATAAATGTTCTAGAACTGTTGATGATAGGCAGTTCTGGTACTGACAGGCCCACCGATTTCTT.

The positive clones were further confirmed by Southern blot analyses using a 3’ probe. DNA was digested with HindIII, and electrophoretically separated on a 0.8% agarose gel. After transfer to a nylon membrane, the digested DNA was hybridized with a probe targeted against the 3’ region (Probe 2):

TGCCTTTCTCTCTGCTCTGTGAGTTAGGGGTGCCTAGGCAGACAGTGAAGAGTACTGTAGCCTTCACTCCCTCCTGTGTGGGTGTGTCCTCTCCTGTCCTGTACTCTGCCATGACAATGAGGCTCTTGTGACAGCCTTTGATTTTAGGCTTTCAAGCAAATCCAAAATACACTAGCGGTAATTCTTTGCCAGGCGTTCTTTATTAGATAAAGTGACGTGAATGGTCTCATGATCAAGTCCCTGCCCATTTGCCTGAACTGACTTAGGTTGGCTCTGTTACTAATGAGCTCTGCTATGTCCACCTGCAGGATGGACGTGGAGAAAGGCAAGATTGAAGACACTGGAGGCAGTGGCGGCTCCTATTCCATCAAAACCCAGTTTAACTCGGAGGAGCAGTGGACAAAAGCGCTCAAGTTCATGCTGACC.

The positive knock-in clones were tested for normal karyotype and used to inject blastocysts from C57BL/6J donors. Mice with germline transmission were bred to mice expressing Cre from the CAG promoter (gift of Eric Olson, UT Southwestern Medical Center) to remove the neomycin cassette. Offspring were genotyped for the presence of the knock-in allele by PCR with the following primers: 5' primer: GGCAGTAGTATAATGTCTGCTCCAG; knock-in 3' primer: TCTAATTCCATCAGAAGCTGACTCT; wild-type 3' primer: TGGGTCTCTCATTGCATTTATTTAT. Using this scheme, the knock-in *Becn1*^F121A^ allele was identified by a PCR product of 650 bp, and the wild-type allele was identified by a PCR fragment of 320 bp. *Becn1*^F121A^ mice were backcrossed for more than 10 generations to C57BL/6J mice (Jackson Laboratories).

For the generation of 5XFAD; *Becn1*^FA/FA^ mice, heterozygous 5XFAD transgenic mice were bred to homozygous *Becn1* knock-in (*Becn1*^FA/FA^) mice to obtain 5XFAD; *Becn1*^FA/+^ mice, which were bred to the *Becn1*^FA/FA^ or *Becn1*^+/+^ littermate mice to produce the 5XFAD; *Becn1*^FA/FA^ and 5XFAD; *Becn1*^+/+^ offspring. For the generation of PDAPP; *Becn1*^FA/FA^ mice, heterozygous PDAPP transgenic mice were bred to homozygous *Becn1* knock-in (*Becn1*^FA/FA^) mice, and the offspring were bred to the *Becn1*^FA/FA^ or *Becn1*^+/+^ littermate mice to produce the PDAPP; *Becn1*^FA/FA^ and PDAPP; *Becn1*^+/+^ mice. Similarly, PDAPP transgenic mice were crossed with homozygous Bcl2^AAA^ mice to produce the PDAPP; Bcl2^AAA^ mice.

### Cell lines

HeLa cell lines were obtained from ATCC, and HeLa cells conditionally expressing CFP-tagged Huntingtin with polyQ repeats were from A. Yamamoto (Columbia University). The HEK293 cell line stably expressing APP (APP-HEK293 cells) was generated by recombinant adenovirus encoding WT human APP under the control of the CMV promoter. Cells were cultured in DMEM medium (Gibco, 11995073) supplemented with 10% FBS. Tetracycline-free FBS was used for HeLa cells stably expressing Huntingtin (Takara Bio USA, 631107), and regular FBS was used for all other cells (HyClone, SH30070.03HI).

### Isolation and culture of primary cortical neurons

Cortical neurons were isolated from E16.5 mouse embryos via dissociation in 0.25% trypsin at 37°C. Neurons from each single embryo were separately plated at the density of 10^5^ cells per well on culture slides (4 well-culture slide) coated with 100 μg/ml poly-L-lysine in borate buffer (50 mM boric acid, 12.5 mM borax). Neurons were plated in neurobasal media (Gibco 21103–049) supplemented with 2% B-27 (Gibco 17504–044), 500 μM glutamine (Invitrogen), 10% horse serum and 2.5 μM glutamate. After 2 h, media was replaced with growth media (neurobasal media with 2% B-27 and 500 μM glutamine).

### Co-immunoprecipitation from muscle and brain tissue

Mouse tissues (muscle and brain) were homogenized in lysis buffer containing 50 mM Tris (pH 7.5), 150 mM NaCl, 1 mM EDTA, 1% Triton X-100, halt proteinase inhibitor cocktail (ThermoFisher Scientific), and halt phosphatase inhibitor cocktail (ThermoFisher Scientific), and subjected to immunoprecipitation with anti-BCL2 monoclonal antibody conjugated agarose beads (Santa Cruz Biotechnology 7382 AC). Eluates were separated by SDS-PAGE and detected by anti-BCL2-HRP antibody (C2 Santa Cruz Biotechnology, 1:500) and anti-BECN1 antibody (Santa Cruz Biotechnology, 1:200) using the ONE-HOUR Western Detection Kit (GenScript Corporation) following the manufacturer’s instruction.

### Autophagosome immunoisolation

Cortex samples from 12-week old 5XFAD; *Becn1*^FA/FA^; GFP-LC3 mice were dissected and homogenized in 1 ml cold lysis buffer pH 7.4 containing 250 mM sucrose, 1 mM EDTA, 10 mM HEPES, halt proteinase inhibitor cocktail (ThermoFisher Scientific), and halt phosphatase inhibitor cocktail (ThermoFisher Scientific), using a Dounce tissue grinder (Wheaton). The lysate was then passed 15 times through 27-gauge needle. GFP-based immunoisolation was performed using Dynabeads Protein G (ThermoFisher Scientific). The lysate was centrifuged at 1,000 x g for 10 min at 4°C. The post-nuclear supernatant fraction was centrifuged at 20,000 x g for 20 min and the supernatant fraction was discarded to remove residual cytosolic GFP-LC3 [61]. The pellet fraction was resuspended in 250 μl lysis buffer and was incubated for 2 hours at 4°C with 40 μl of Dynabeads, preincubated O/N with GFP-antibody (Sigma, G1544). The beads were then washed 4 times with wash buffer (150 mM NaCl, 250 mM sucrose, 1 mM EDTA, 10 mM HEPES) using the magnetic Separator DynaMag^TM^-2 (ThermoFisher Scientific). Immunoprecipitates were eluted with lysis buffer containing 1X sample buffer and analyzed by SDS-PAGE.

### Drug treatment

For in vivo use, ML246 was dissolved in a solvent containing 5% of NMP, 20% of PEG400 and 75% of 10% HP-β-CD in water, and injected intraperitoneally at the dosage of 5 mg/kg body weight for 5 weeks, 5 days per week. To measure the autophagy flux in vivo, chloroquine was dissolved in PBS and injected intraperitoneally at the dosage of 50 mg/kg, To inhibit autophagy in vivo, SBI-0206965 (Adooq Bioscience; A15795) was dissolved in PBS containing 50% DMSO, and injected intraperitoneally into mice at the dosage of 2 mg/kg body weight once per day for 7 days. Mice were sacrificed and tissues were collected 4 h after the last drug injection. For cell culture use, ML246 was dissolved in 100% DMSO and used at the concentration of 0.5 μM.

### Mouse exercise studies

For acute exercise studies, 8-week old mice (wild-type and *Becn1*^FA/FA^ mice crossed to GFP-LC3 transgenic mice) were acclimated and trained on a 10° uphill Exer 3/6 open treadmill (Columbus Instruments) for 2 days. On day 1 mice ran for 5 min at 8 m/min and on day 2 mice ran for 5 min at 8 m/min followed by another 5 min at 10 m/min. On day 3, mice were subjected to a single bout of running starting at the speed of 10 m/min. Forty minutes later, the treadmill speed was increased at a rate of 1m/min every 10 min for 30 min, and then increased at rate of 1 m/min every 5 min for 20 min, so that the mice ran for a total of 90 minutes of exercise and 1070 meters of running distance. For long-term exercise, 2-month old 5XFAD mice were single-housed in a cage containing a running wheel (11.4 cm diameter) for a total of 4 months. The running capacity of mice was monitored by an odometer connected to the wheel.

### Morris water maze testing

For animal behavior, 6-month old mice were tested. The Morris water maze test consists of two sections: the visible platform testing and hidden platform testing. During the tests, mice were placed in the water tank filled with opaque water (maintained at 25°C), with their heads facing toward the tank wall. In the visible platform section, a black platform extending 2 cm above the water level was used for these trials. For each trial, the platform was randomly positioned, and the mouse was placed in the tank at different start positions. The trial was stopped after the mouse found and climbed onto the platform, and the escape latency was recorded. The trial was stopped if the mouse did not climb onto the platform in 60 s, and the experimenter guided it to the platform. Mice were tested for 4 days with eight trials per day. In the hidden platform section, a transparent platform underneath the water level was used instead of the black one during all trials, mice were tested with a fixed platform location over 5 days period with six trials per day, and they were allowed to search the platform in 60 s. In the tests, two parameters were evaluated: the trail duration (s) and distance to the platform (m).

### Dot blot assay

Snap frozen hemi-brain were homogenized in 800 μl phosphate-buffered saline (PBS; Sigma-Aldrich, D8537) with 1% Triton X-100 supplemented with halt proteinase inhibitor cocktail (ThermoFisher Scientific), and halt phosphatase inhibitor cocktail (ThermoFisher Scientific). Protein concentration was quantified using BCA Assay (Pierce). For Aβ42 dot blots, 10 mg/ml brain homogenates were extracted in guanidine buffer (5 M guanidine-HCl [GuHCl], 50 mM Tris HCl pH 8.0) overnight at room temperature. One μl of sample was spotted in triplicate on 0.2 μm nitrocellulose membrane, and dried for 1 h at 37°C. The membrane was stained with Ponceau S, and the dot blot signal on the membrane was detected by immunostaining with Aβ42 antibody (Invitrogen, 700254, 1:1000) and HRP-conjugated secondary antibody (Santa Cruz Biotechnology, sc2004, 1:2000). Aβ42 signals were normalized to the Ponceau S staining. To separate soluble and insoluble Aβ fractions, 10 mg/ml of the total homogenated brains were centrifuged at 14000 rpm, 4°C for 30 min. The supernatant (soluble fraction) was used directly for dot blot assays. The pellet (insoluble fraction) was extracted in guanidine buffer overnight at room temperature, and used in dot blot analyses. To measure Aβ levels in conditioned media of APP-HEK293 cells, media of 72-h cell culture was collected, mixed with 4X sample buffer (50 mM Tris-HCl pH6.8, 2% SDS, 10% glycerol, 1% β-mercaptoethanol, 12.5 mM EDTA, 0.02% bromophenol blue), and boiled at 95°C for 10 min. One μl of each sample was spotted on nitrocellulose membrane for dot blot analysis.

### ELISA

GuHCl extracted brain samples prepared in the same way as dot blot assays were diluted 1:1000, and ELISA analyses of Aβ42 were performed according to manufacturer’s instructions (Thermo Fisher Scientific, KHB3441).

### Immunofluorescence microscopy

Paraformaldehyde-fixed brain tissues were sectioned at 30 μm thickness. Free-floating sagittal sections were immnunostained with 1% thioflavin S (Sigma-Aldrich, 230456) for 20 minutes. Additional sections were immunostained with Aβ antibody (Invitrogen, 700254, 1:500) and Alexa Fluro 594 goat anti rabbit (ThermoFisher Scientific, A11012). Sections were mounted on slides with mounting medium containing DAPI (Vectashield) and then analyzed by fluorescence microscopy under the 10x objective.

### Confocal microscopy

Cortical neurons derived from PDAPP *Becn1*^+/+^ and PDAPP *Becn1*^FA/FA^ embryos were grown on poly-L-lysine coated culture slides. Cells (9 DIV) were then fixed in 4% paraformaldehyde and permeabilized with 0.3% Triton X-100. Slides were blocked for 1 h in PBS containing 1% BSA and 2% normal goat serum and then incubated overnight at 4°C with primary antibodies: anti-APP (Biolegend; 803001) and anti-Rab5 (Cell Signaling Technology; 3547) or anti-Rab7 (Cell Signaling Technology; 9367). After washing, slides were incubated with species-specific Alexa-dye conjugated secondary antibodies for 1 h at room temperature. Slides were sealed with coverslip using mounting medium containing DAPI (Vectashield) and then analyzed by confocal microscopy. Confocal images were collected on Nikon A1 microscope using a 60x oil immersion objective lens and NIS Elements software. The Mander’s colocalization coefficient and the fluorescence intensity profile were generated using the NIS Element software.

### Autophagy analyses

For assessment of autophagy in vivo, 8-week old male WT and *Becn1*^FA/FA^ mice crossed to GFP-LC3 mice were exercised for 90 minutes, or starved for 48 h, and then anaesthetized by isoflurane and perfused with 4% PFA. Brain and muscle samples were fixed in 4% PFA overnight, 15% sucrose for 4 h and 30% sucrose overnight before frozen sections were prepared. The number of GFP-LC3 puncta per unit area of tissue was quantified by fluorescence microscopy. Autophagy in vivo was also analyzed by western blot analysis of brain tissue extracts with antibodies against LC3 and p62/SQSTM1 (see below for details).

### Western blot analyses

Cell or mouse muscle and brain extracts were prepared in lysis buffer containing 50 mM Tris (pH 7.4), 150 mM NaCl, 1 mM EDTA, 1% Triton X-100, halt proteinase inhibitor cocktail (ThermoFisher Scientific) and halt phosphatase inhibitor cocktail (ThermoFisher Scientific), and subjected to western blot analysis with anti-LC3 (Novus Biologicals, NB100-2220), anti-SQSTM1 (Abnova, H00008878-M01), anti-Aβ42 (Invitrogen; 700254), anti-APP (Biolegend; 803001), HRP-conjugated GFP antibody (Santa Cruz Biotechnology, sc9996), anti-HA (Cell Signaling Technology, C29F4), anti-ATG7 (Sigma Aldrich, A2856), anti-LDLR (Abcam, ab52818), anti-LRP1 (Abcam, ab92544), and anti-ACTB/β-actin-HRP (Santa Cruz Biotechnology, sc47778 HRP) antibodies. The band intensity was analyzed using the ImageJ software.

### Filter trap assay

HeLa cells stably expressing CFP-HTT25Q, CFP-HTT65Q and CFP-HTT103Q were treated with 100 ng/ml tetracycline (IBI Scientific, IB02200) for 48 h, or 0.5 μM ML246 for 24 h with control or *ATG7* siRNA (GE Dharmacon ON-TARGETplus control or *ATG7* SMARTpool siRNA) for 48 h. Cells were then collected and lysed in lysis buffer containing 0.5% NP-40 at 4°C for 30 min. After centrifugation, the pellet was digested with 0.5 mg/ml DNaseI (in 20 mM Tris-Cl, pH 8.0) for 1 h at 37°C, and dissolved into lysates containing insoluble aggregates by 2% SDS, 50 mM DTT and 20 mM EDTA. The lysates were then added onto 0.2 μm nitrocellulose membrane that was pre-equilibrated with 2% SDS/TBS for 30 min, and were filtered through the membrane by gentle vacuum using the Bio-Dot SF microfiltration apparatus (Bio-Rad). The signal was detected by immunostaining with the HRP-conjugated GFP antibody (Santa Cruz Biotechnology, sc9996, 1:1000).

### TUNEL assay

APP-HEK293 cells were cultured in 6-well dishes for 24 h and then the media was replaced with neuronal culture media. After 48 h, conditioned media was collected and used to treat primary cortical neurons (12 DIV) cultured on poly-L-lysine coated slides for another 24 h. Apoptotic neurons were detected by the In Situ Cell Death Detection Kit, TMR red (Roche, cat. # 12156792910) according to the manufacturer’s instructions. Nuclei were stained using the mounting medium containing DAPI (Vectashield). Quantification of red TUNEL-positive neurons was done using the NIS Elements software.

### Generation of *BECN1* knock out cells by CRISPR/Cas9

The gRNA sequence against human *BECN1* genome in exon1 (5’-ggacacgagtttcaagatcctgg-3’; underline indicates the protospacer adjacent motif) was designed using the CRISPR Design tool (http://crispr.mit.edu:8079) [62], which contained a Sau3AI restriction enzyme site at the Cas9 cutting position on its gRNA sequence. Annealed oligonucleotides were inserted into the pSpCas9(BB)-2A-puro (PX459) V2.0 vector (Addgene, #62988). The plasmid was transfected into APP-HEK293 cells using lipofectamine 3000 (Thermo Fisher Sciencetific), and cells were selected for 72 h using DMEM supplemented with 2 μg/ml puromycin. Genome editing efficiency and protein expression levels were confirmed by Sau3AI enzymatic digestion and western blotting, respectively.

### Generation of *Becn1*^F121A^-expressing stable cells

Mouse wild-type (WT) *Becn1* or *Becn1*^F121A^ mutant cDNA was sub-cloned into the pCDH-CMV-MCS-EF1-GreenPuro vector (System Biosciences, Palo Alto, CA, USA) using XbaI and BamHI restriction sites. Lentivirus encoding *Becn1* or *Becn1*^F121A^ was produced by co-transfection of packing plasmids, pCMV-VSV-G (Addgene, #8454) and psPAX2 (Addgene, #12260) into HEK293 FT cells. The resulting lentivirus encoding *Becn1* or *Becn1*^F121A^ was used to infect *BECN1* KO cells at the multiplicity of infection of 1 for 24 h in the presence of 10 μg/ml polybrene (Santa Cruz Biotechnology). Infected cells were selected and maintained in DMEM supplemented with 2 μg/ml puromycin (Thermo Fisher Scientific).

### Biotin protection assay on cell-surface APP trafficking

The biotinylation procedure was modified from a previously reported protocol [63]. HEK293 cells stably expressing APP were grown to 90% confluency on gelatin-coated 6 cm^2^ dish, washed with ice-cold PBS, and incubated in 0.3 mg/ml disulfide-cleavable biotin (EZlink Sulfo-NHS-SS-Biotin, Thermo Scientific) in PBS at 4°C for 30 min. Cells were then washed with cold PBS and returned to warm medium at 37°C, and incubated for 5 or 15 min. Cells labeled “Total” were left on ice in PBS. Cells labeled “Stripping” were also left on ice in PBS and then stripped as described below. The remaining cell-surface biotinylated APP was stripped in 50 mM glutathione, 0.3 M NaCl, 75 mM NaOH, 10% FBS at 4°C for 40 min. Glutathione was then quenched with 50 mM iodoacetamide and 1% bovine serum albumin in PBS at 4°C for 15 min. Proteins were extracted in lysis buffer containing 0.1% sodium dodecyl sulfate (SDS), 0.5% sodium deoxycholate, 1% Triton X-100, 100 mM NaCl, 2 mM EDTA and 50 mM Tris-HCl 7.4 supplemented with protease and phosphatase inhibitor cocktail (Thermo Scientific), and supernatant was collected by centrifugation at 10,000 xg for 10 min at 4°C. Biotinylated APP were isolated using streptavidin-agarose (Millipore) at 4°C for 2 h. Precipitates were washed four times with wash buffer containing 0.1% SDS, 1% Triton X-100, 100 mM NaCl, 2 mM EDTA and 50 mM Tris-HCl 7.4, and proteins were eluted in SDS sample buffer by boiling.

### Statistical analyses

P ≤ 0.05 was considered statistically significant in two-tailed, unpaired Student’s t-tests for detection of differences between two experimental groups; Two-way ANOVA approach was used for comparison among multiple groups. Statistics on the survival study was done by the log-rank test. Figures are depicted as mean ± SEM.

## Supporting information

S1 Fig Generation of *Becn1*^F121A^ knock-in mice. **(A)** Genomic structure of *Becn1* and the *Becn1* F121A knock-in targeting vector. **(B)** Southern blot analyses of genomic DNA from *Becn1*^+/+^ and *Becn1* ^F121A/+^ embryonic stem cells, using the two probes listed in (A) and Methods. **(C)** Genotyping of F2 pups by PCR using primers listed in Methods.
(TIF)

S2 Fig The knock-in *Becn1*^F121A^ mutant expresses at a similar level as WT Becn1 in vivo. Western blot analyses (left) and quantification (right) of *Becn1* in the indicated tissues from WT mice and *Becn1*^F121A^ knock-in mice. Results represent mean ± s.e.m. NS, not significant; t-test.
(TIF)

S3 Fig Higher autophagy flux in *Becn1*^FA/FA^ mice. Representative images (upper panel) and flux quantification (lower panel) of GFP-LC3 puncta (autophagosomes) in skeletal muscle of GFP-LC3 *Becn1*^+/+^ and GFP-LC3 *Becn1*^FA/FA^ mice injected with one dose of PBS or 50 mg/kg lysosomal inhibitor chloroquine at non-autophagy-inducing conditions. The autophagy flux is measured by the difference in the number of GFP-LC3 puncta between mice injected with PBS and with chloroquine. Results represent mean ± s.e.m. N = 5. **, P<0.01, t-test.
(TIF)

S4 Fig APP expression is not affected by the *Becn1*^FA/FA^ mutation, ML246 treatment, or exercise. **(A)** Western blot analysis (left) and quantification (right) of APP in brain samples of 5XFAD mice expressing WT *Becn1* or *Becn1*^F121A^. **(B)** Western blot analysis (left) and quantification (right) of APP in brain of 5XFAD mice treated with vehicle or ML246 for 5 weeks. **(C)** Western blot analysis (left) and quantification (right) of APP in brain of 5XFAD mice housed under normal conditions or subject to 4 months of voluntary running. A WT mouse without APP transgene was used as negative control. 4–6 mice were used for each group. Results represent mean ± s.e.m. NS, not significant; t-test.
(TIF)

S5 Fig The effect of *Becn1*^F121A^ on amyloid metabolism is dependent on the autophagy pathway. **(A)** Western blot analysis and quantification of intracellular Aβ42 in HEK293 cells stably expressing *APP* and *Becn1*^F121A^. Control or *ATG7* siRNA was transfected 48 h prior to cell lysis. N = 4 independent experiments. **(B)** Immunofluorescence imaging and quantification of colocalization between APP and endosomal markers Rab5 or Rab7 in primary cortical neurons of PDAPP *Becn1*^+/+^ mice or PDAPP *Becn1*^FA/FA^ mice. The level of colocalization between APP and Rabs was quantified by Mander's overlap coefficient. **(C)** Biotin protection assays on biotin-labeled internalized APP by streptavidin affinity isolation in *BECN1* KO HEK293 cells stably expressing APP and HA-*Becn1* or HA-*Becn1*^F121A^. Cell-surface total APP was biotinylated at 4°C (“Total”), and the level of APP internalization was analyzed by protection from glutathione stripping after incubation at 37°C for 5 min (“5 min”) or 15 min (“15 min”). As control, cells immediately treated with glutathione stripping (“Stripping”) without inducing endocytosis at 37°C showed barely detectable APP biotinylation. Expression of HA-*Becn1* or HA-*Becn1*^F121A^ was shown on the left. N = 4 independent experiments. **(D)** Western blot analysis and quantification of LRP1 and LDLR in brain samples of 5XFAD mice expressing WT *Becn1* or *Becn1*^F121A^. N = 6. Results represent mean ± s.e.m. NS, not significant; *, P<0.05; t test.
(TIF)

S6 Fig The autophagy inducer ML246 decreases polyglutamine aggregates in an autophagy-dependent manner in cells. **(A)** Filter trap assay (upper) and quantification (lower) of stable HeLa cells conditionally expressing HTT25Q-CFP, HTT65Q-CFP or HTT103Q-CFP in a Tet-off system, in the presence or absence of ML246 or the indicated siRNA. Cells were transfected with non-targeting control (NC) or *ATG7* siRNA 24 h prior to ML246 treatment for another 24 h. HTT aggregates were analyzed by lysate filtration through 0.2 μm nitrocellulose membrane. Cells treated with tetracycline served as negative control. **(B)** Representative images (upper) and quantification (lower) of inclusions formed by CFP-tagged polyglutamine HTT in cells as in (A). Blue, DAPI. Results represent mean ± s.e.m. Scale bar: 20 μm. Statistics compare each value to the one under the “-” condition. *, P<0.05; **, P<0.01; ***, P<0.001, t test.
(TIF)

S7 Fig Hyperactivation of autophagy by *Becn1*^F121A^, ML246 or voluntary exercise ameliorates cerebral Aβ plaques accumulation in 5XFAD mice. **(A)** Representative images (upper) and quantification (lower) of amyloid deposits stained by anti-Aβ42 antibody in brain of 6-month old 5XFAD mice, 5XFAD *Becn1*^FA/FA^ mice, and 5XFAD mice subject to 5 weeks of ML246 treatment or 4 months of voluntary exercise. Scale bar: 500 μm. Results represent mean ± s.e.m. N = 6–8. *, P<0.05; **, P<0.01, t test. **(B)** Working model of autophagic degradation of Aβ42 in AD brain: neurons (yellow) degrade de-novo processed Aβ via autophagy, whereas glial cells (purple) re-uptake and degrade neuronal-secreted Aβ42 from the extracellular space.
(TIF)
