# Supplementary figures and images for: A Becn1 mutation mediates hyperactive autophagic sequestration of amyloid oligomers and improved cognition in Alzheimer's disease
Source: PLoS Genet. 2017 Aug 14;13(8):e1006962. doi: 10.1371/journal.pgen.1006962 (PMC5570506; doi:10.1371/journal.pgen.1006962)

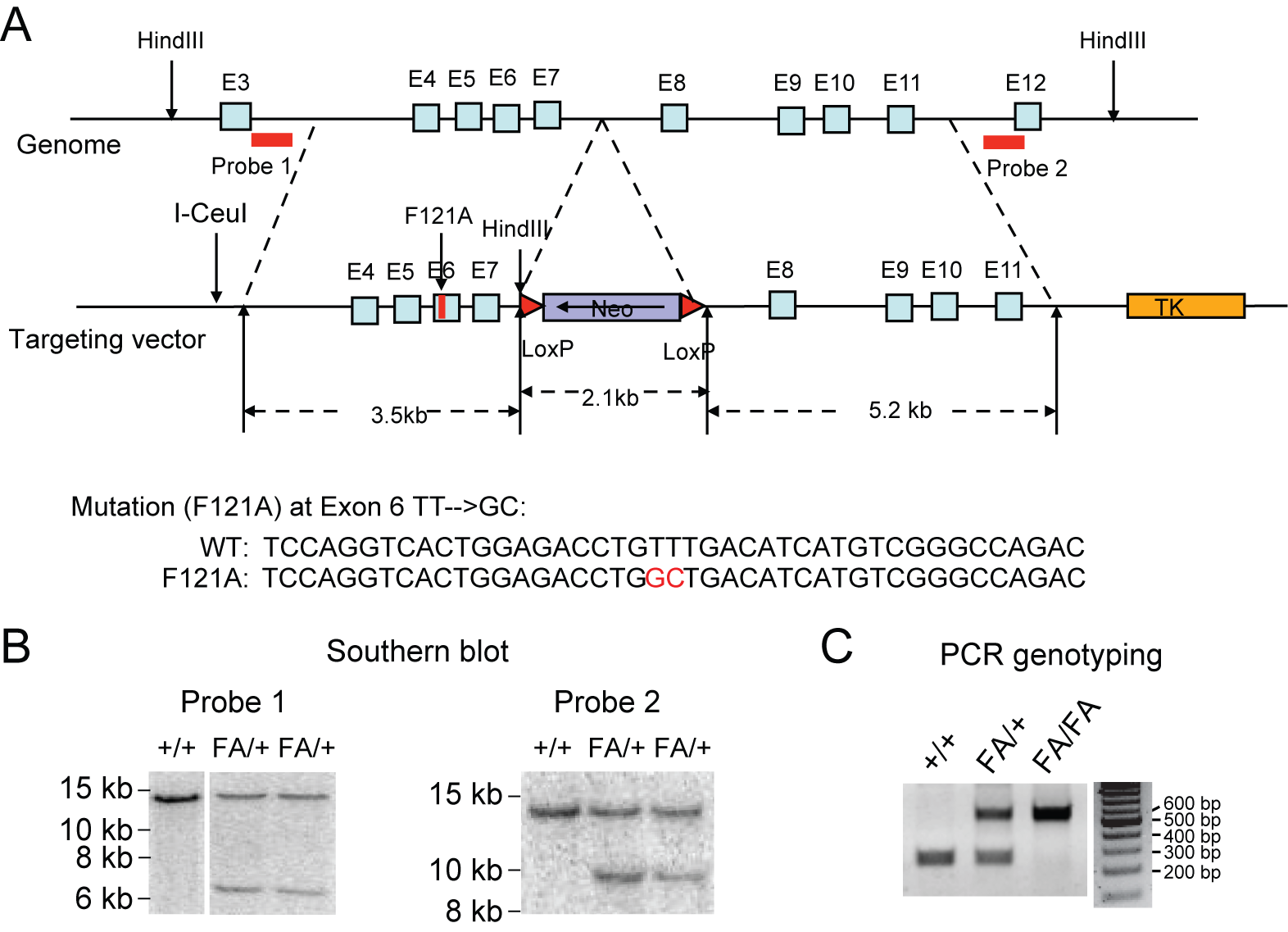

Supplement: S1 Fig — (A) Genomic structure of Becn1 and the Becn1 F121A knock-in targeting vector. (B) Southern blot analyses of genomic DNA from Becn1+/+ and Becn1 F121A/+ embryonic stem cells, using the two probes listed in (A) and Methods. (C) Genotyping of F2 pups by PCR using primers listed in Methods. (TIF) [file pgen.1006962.s001.tif]

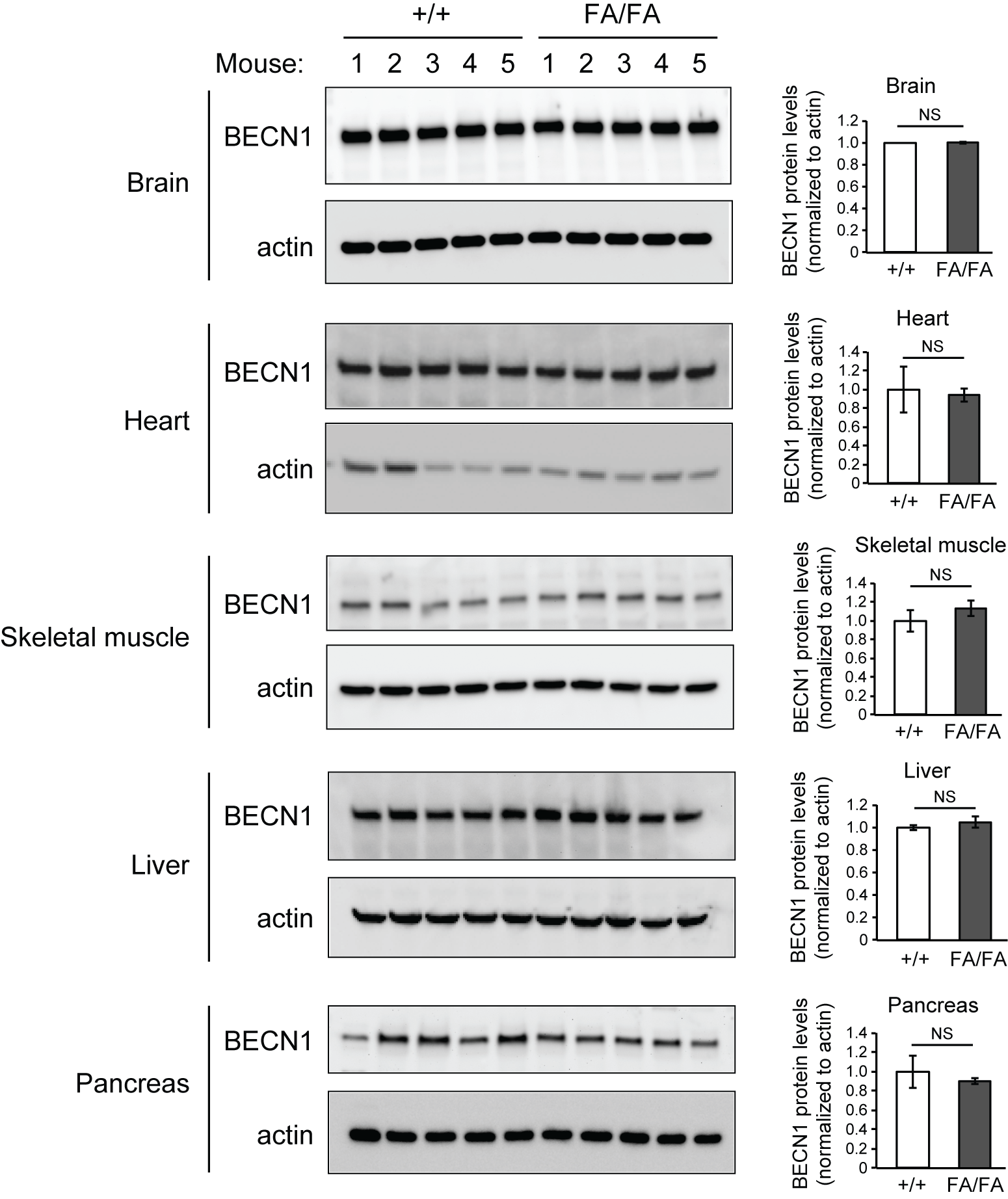

Supplement: S2 Fig — Western blot analyses (left) and quantification (right) of Becn1 in the indicated tissues from WT mice and Becn1F121A knock-in mice. Results represent mean ± s.e.m. NS, not significant; t-test. (TIF) [file pgen.1006962.s002.tif]

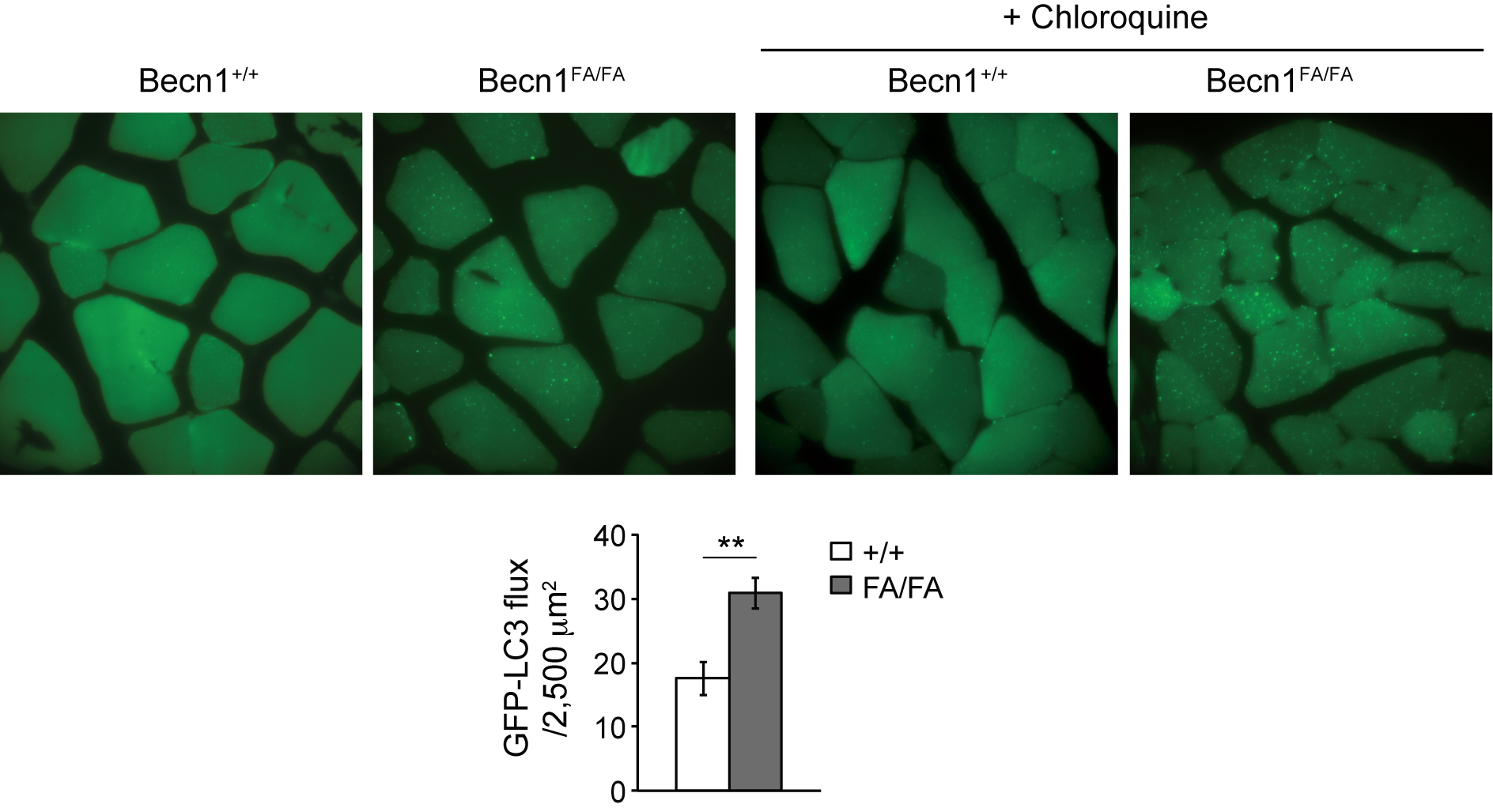

Supplement: S3 Fig — Representative images (upper panel) and flux quantification (lower panel) of GFP-LC3 puncta (autophagosomes) in skeletal muscle of GFP-LC3 Becn1+/+ and GFP-LC3 Becn1FA/FA mice injected with one dose of PBS or 50 mg/kg lysosomal inhibitor chloroquine at non-autophagy-inducing conditions. The autophagy flux is measured by the difference in the number of GFP-LC3 puncta between mice injected with PBS and with chloroquine. Results represent mean ± s.e.m. N = 5. **, P<0.01, t-test. (TIF) [file pgen.1006962.s003.tif]

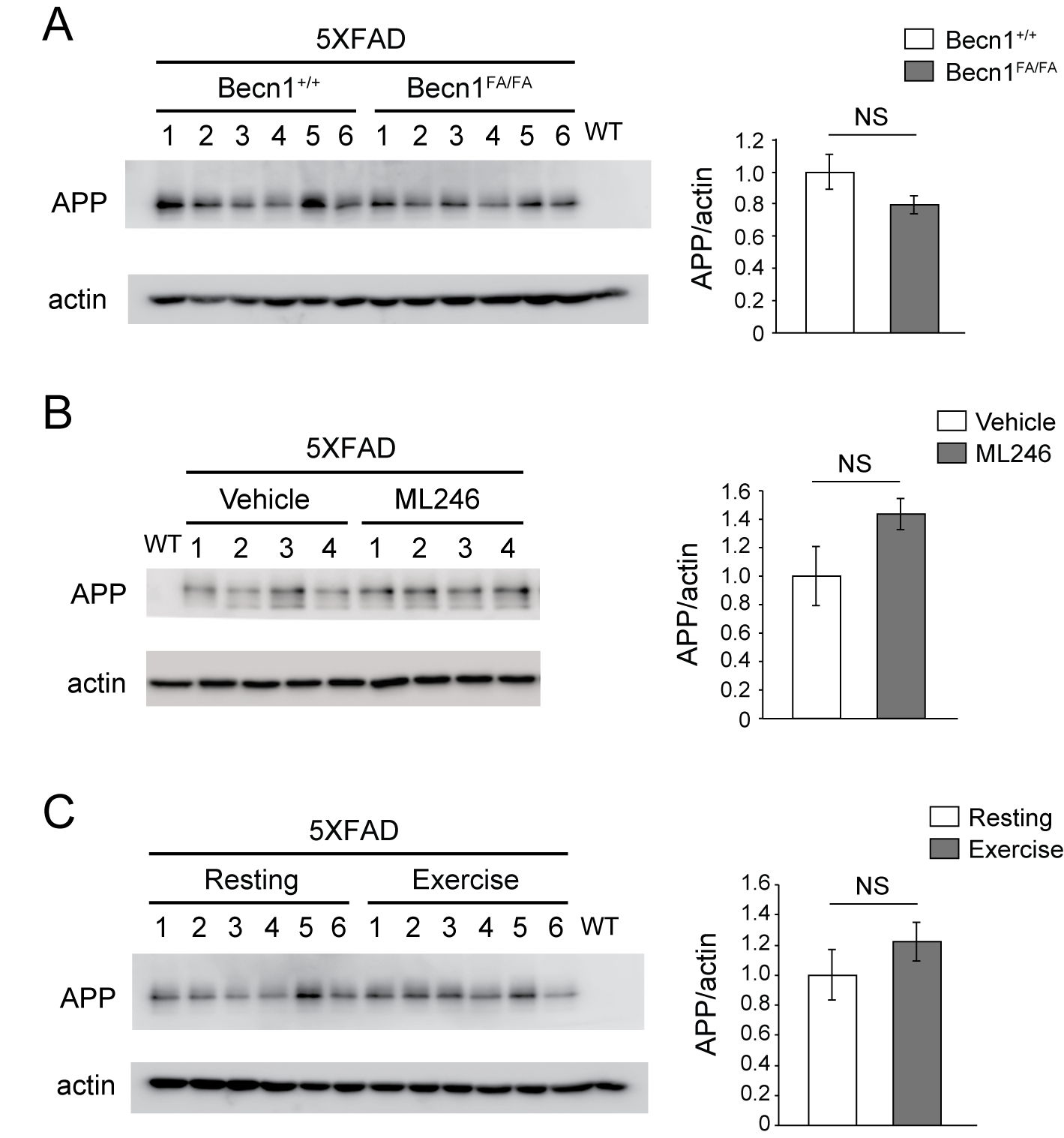

Supplement: S4 Fig — (A) Western blot analysis (left) and quantification (right) of APP in brain samples of 5XFAD mice expressing WT Becn1 or Becn1F121A. (B) Western blot analysis (left) and quantification (right) of APP in brain of 5XFAD mice treated with vehicle or ML246 for 5 weeks. (C) Western blot analysis (left) and quantification (right) of APP in brain of 5XFAD mice housed under normal conditions or subject to 4 months of voluntary running. A WT mouse without APP transgene was used as negative control. 4–6 mice were used for each group. Results represent mean ± s.e.m. NS, not significant; t-test. (TIF) [file pgen.1006962.s004.tif]

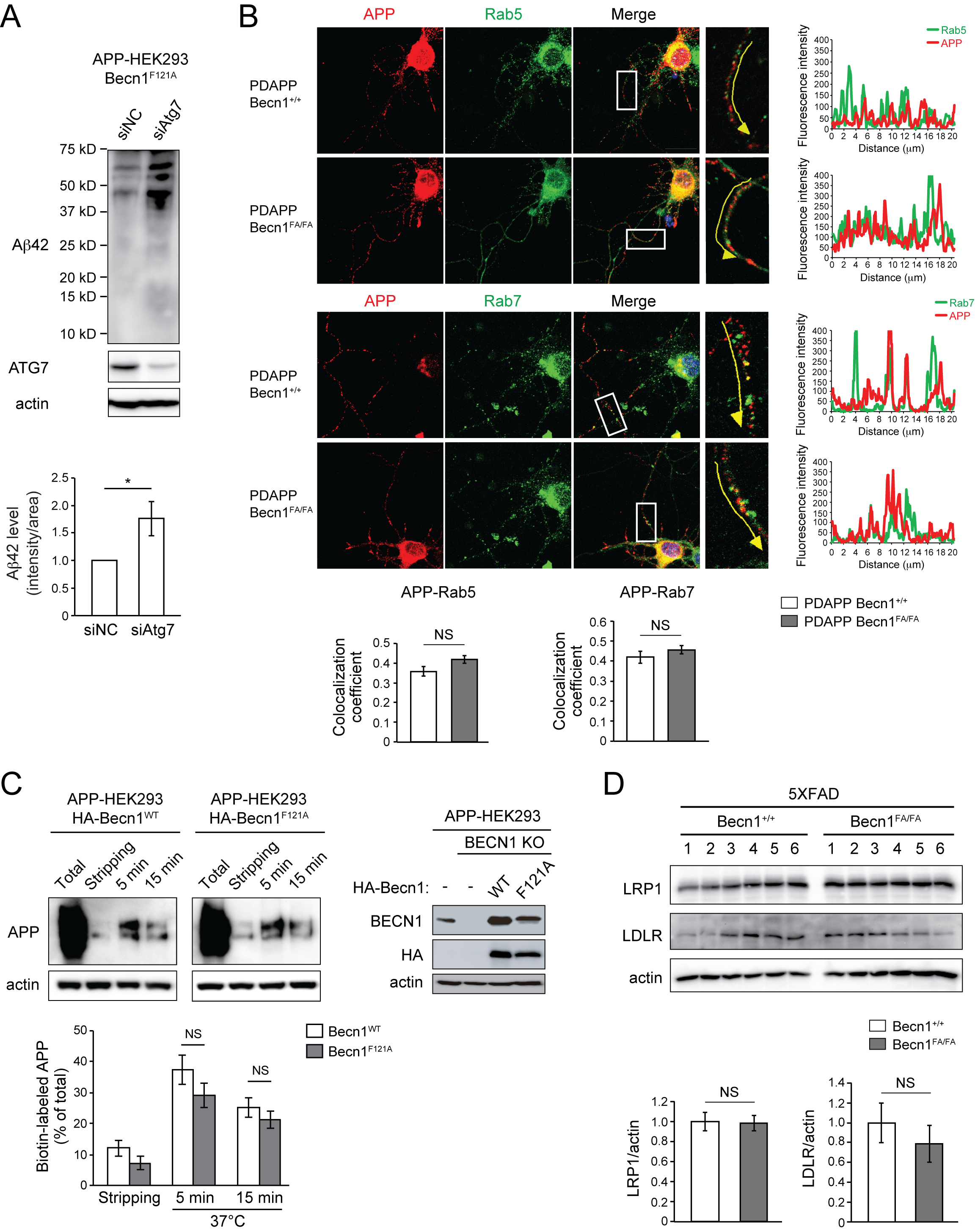

Supplement: S5 Fig — (A) Western blot analysis and quantification of intracellular Aβ42 in HEK293 cells stably expressing APP and Becn1F121A. Control or ATG7 siRNA was transfected 48 h prior to cell lysis. N = 4 independent experiments. (B) Immunofluorescence imaging and quantification of colocalization between APP and endosomal markers Rab5 or Rab7 in primary cortical neurons of PDAPP Becn1+/+ mice or PDAPP Becn1FA/FA mice. The level of colocalization between APP and Rabs was quantified by Mander's overlap coefficient. (C) Biotin protection assays on biotin-labeled internalized APP by streptavidin affinity isolation in BECN1 KO HEK293 cells stably expressing APP and HA-Becn1 or HA-Becn1F121A. Cell-surface total APP was biotinylated at 4°C (“Total”), and the level of APP internalization was analyzed by protection from glutathione stripping after incubation at 37°C for 5 min (“5 min”) or 15 min (“15 min”). As control, cells immediately treated with glutathione stripping (“Stripping”) without inducing endocytosis at 37°C showed barely detectable APP biotinylation. Expression of HA-Becn1 or HA-Becn1F121A was shown on the left. N = 4 independent experiments. (D) Western blot analysis and quantification of LRP1 and LDLR in brain samples of 5XFAD mice expressing WT Becn1 or Becn1F121A. N = 6. Results represent mean ± s.e.m. NS, not significant; *, P<0.05; t test. (TIF) [file pgen.1006962.s005.tif]

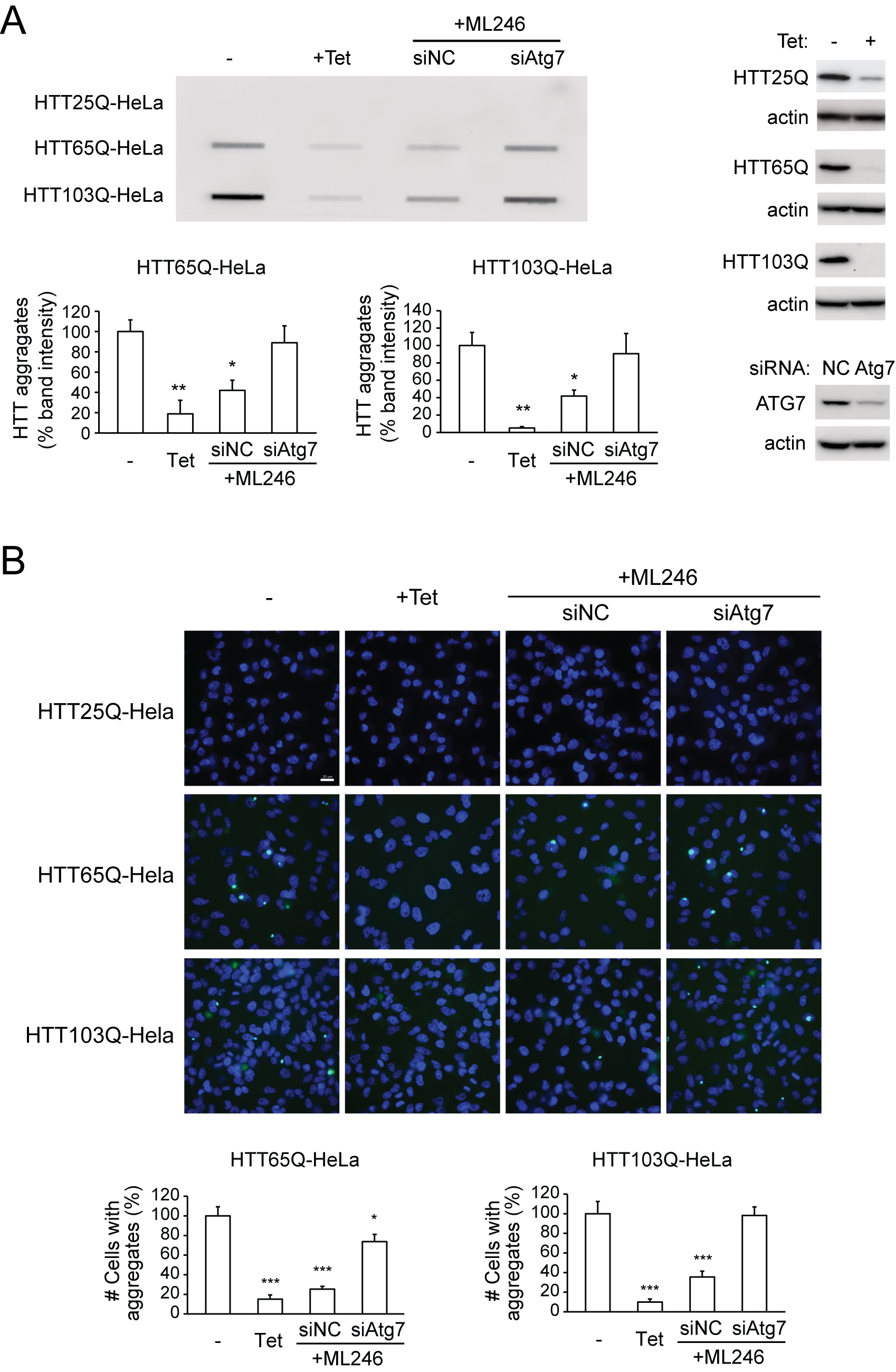

Supplement: S6 Fig — (A) Filter trap assay (upper) and quantification (lower) of stable HeLa cells conditionally expressing HTT25Q-CFP, HTT65Q-CFP or HTT103Q-CFP in a Tet-off system, in the presence or absence of ML246 or the indicated siRNA. Cells were transfected with non-targeting control (NC) or ATG7 siRNA 24 h prior to ML246 treatment for another 24 h. HTT aggregates were analyzed by lysate filtration through 0.2 μm nitrocellulose membrane. Cells treated with tetracycline served as negative control. (B) Representative images (upper) and quantification (lower) of inclusions formed by CFP-tagged polyglutamine HTT in cells as in (A). Blue, DAPI. Results represent mean ± s.e.m. Scale bar: 20 μm. Statistics compare each value to the one under the “-” condition. *, P<0.05; **, P<0.01; ***, P<0.001, t test. (TIF) [file pgen.1006962.s006.tif]

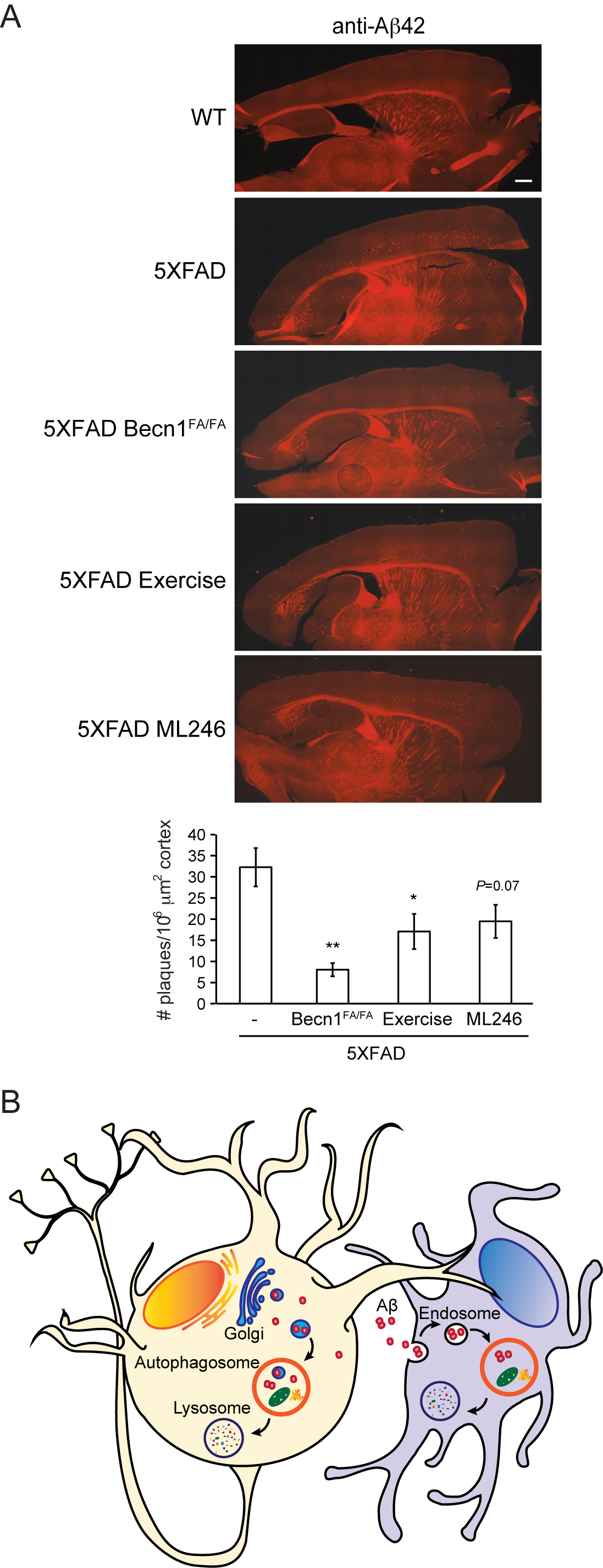

Supplement: S7 Fig — (A) Representative images (upper) and quantification (lower) of amyloid deposits stained by anti-Aβ42 antibody in brain of 6-month old 5XFAD mice, 5XFAD Becn1FA/FA mice, and 5XFAD mice subject to 5 weeks of ML246 treatment or 4 months of voluntary exercise. Scale bar: 500 μm. Results represent mean ± s.e.m. N = 6–8. *, P<0.05; **, P<0.01, t test. (B) Working model of autophagic degradation of Aβ42 in AD brain: neurons (yellow) degrade de-novo processed Aβ via autophagy, whereas glial cells (purple) re-uptake and degrade neuronal-secreted Aβ42 from the extracellular space. (TIF) [file pgen.1006962.s007.tif]
